# Mechanisms of uropathogenic *E. coli* mucosal association in the gastrointestinal tract

**DOI:** 10.1126/sciadv.adp7066

**Published:** 2025-01-31

**Authors:** Philippe N. Azimzadeh, George M. Birchenough, Nathaniel C. Gualbuerto, Jerome S. Pinkner, Kevin O. Tamadonfar, Wandy Beatty, Thomas J. Hannan, Karen W. Dodson, Enid C. Ibarra, Seonyoung Kim, Henry L. Schreiber, James W. Janetka, Andrew L. Kau, Ashlee M. Earl, Mark J. Miller, Gunnar C. Hansson, Scott J. Hultgren

**Affiliations:** ^1^ Department of Molecular Microbiology, Washington University in St. Louis, School of Medicine, St. Louis, MO 63110, USA.; ^2^ Department of Medical Biochemistry and Cell Biology, Institute of Biomedicine, University of Gothenburg, Gothenburg, Sweden; Wallenberg Centre for Molecular and Translational Medicine, University of Gothenburg, Gothenburg, Sweden.; ^3^ Department of Pathology and Immunology, Washington University School of Medicine, St. Louis, MO 63110, USA.; ^4^ Department of Internal Medicine, Division of Infectious Diseases, Washington University School of Medicine, St. Louis, MO 63110, USA.; ^5^ Center for Women’s Infectious Disease Research, Washington University School of Medicine, St. Louis, MO, USA.; ^6^ Department of Biochemistry and Molecular Biophysics, Washington University in St. Louis, St. Louis, MO 63110, USA.; ^7^ Division of Allergy and Immunology, Department of Medicine, Washington University School of Medicine, St. Louis, MO 63110, USA.; ^8^ Infectious Disease and Microbiome Program, Broad Institute, Cambridge, MA 02142, USA.; ^9^ Department of Medical Biochemistry and Cell Biology, Institute of Biomedicine, University of Gothenburg, Gothenburg, Sweden.

## Abstract

Urinary tract infections (UTIs) are highly recurrent and frequently caused by Uropathogenic *Escherichia coli* (UPEC) strains that can be found in patient intestines. Seeding of the urinary tract from this intestinal reservoir likely contributes to UTI recurrence (rUTI) rates. Thus, understanding the factors that promote UPEC intestinal colonization is of critical importance to designing therapeutics to reduce rUTI incidence. Although *E. coli* is found in high abundance in large intestine mucus, little is known about how it is able to maintain residence in this continuously secreted hydrogel. We discovered that the FimH adhesin of type 1 pili (T1P) bound throughout the secreted mucus layers of the colon and to epithelial cells in mouse and human samples. Disruption of T1P led to reduced association with colon mucus. Notably, this mutant up-regulated flagellar production and infiltrated the protective inner mucus layer of the colon. This could explain how UPEC resists being washed off by the continuously secreted mucus layers of the colon.

## Teaser

Inverse regulation of flagella and type 1 pili facilitates access to colon mucus by uropathogenic *E. coli*.

## INTRODUCTION

Urinary tract infections (UTIs) are common, highly recurrent, and a leading cause of antibiotic therapy among otherwise healthy adult women, accounting for 15% of all outpatient antibiotic prescriptions (*1*–*4*). Uropathogenic *Escherichia coli* (UPEC) causes more than 80% of community-acquired UTIs (*2*). When untreated, UPEC can ascend the ureters to the kidney and access the bloodstream causing sepsis, with up to 20% of sepsis cases resulting from infection by a urinary isolate (*5*). Even when infection is cleared from the urine by clinical antibiotic regimens, 27% of women will experience recurrent UTI (rUTI) within months of an initial UTI (*2*). Greater than 60% of rUTIs are caused by the same strain of *E. coli* that caused the initial infection (*2*, *6*), suggesting that one or more host-associated reservoirs of UPEC can reinoculate the urinary tract in susceptible women. The intestine is one such reservoir, with its resident microbiota acting as a major reservoir from which UPEC can be shed to seed colonization of the lower urogenital tracts and urinary tract (*6*). However, the mechanisms underpinning UPEC colonization and persistence within the gut are not fully understood. A better understanding of these mechanisms, including how UPEC strains colonize specific habitats of the gastrointestinal tract could inform the design of therapeutics to reduce the rate of rUTI incidence.

Colon epithelium is protected from direct contact with bacteria by the secreted mucus hydrogel organized around the Muc2 mucin (*7*). The thickness and barrier function provided by this hydrogel increases along the length of the intestinal tract, as does the number of luminal bacteria. In the colon, the inner mucus layer (IML) is anchored to crypt goblet cells, which continuously secrete mucus. The IML is transformed by endogenous proteolytic activity into the outer loose mucus layer (OML), expanding about three times in volume during this process. The OML is the habitat of a vast population of microbial flora, while the IML is generally devoid of bacteria during homeostasis (*7*–*9*).

Previous studies suggest that some *E. coli* strains preferentially reside in the large intestine, where they are often found in the mucus layers rather than in direct contact with the host (*10*, *11*). In some cases, *E. coli* has also been found to reside within the colonic crypts (*12*). UPEC isolates carry an arsenal of proteinaceous, adhesive fibers, called chaperone usher pathway (CUP) pili, each of which is tipped with an adhesin thought to confer binding specificity to host glycans (*13*). The pangenome of *E. coli* encodes 38 distinct CUP pilus types and single *E. coli genomes* encode as many as 16 distinct, intact CUP operons, each likely mediating colonization of a particular host or environmental habitat (*13*). Each operon encodes structural pilin subunits, a periplasmic chaperone, and an outer membrane usher assembly protein (*14*). These operons also encode a two-domain tip adhesin, consisting of an N-terminal receptor binding domain, which binds with stereochemical specificity to a host or environmental receptor, and a C-terminal pilin domain, which links the adhesin to the rest of the pilus (*14*). On the basis of usher phylogeny, CUP pili are categorized into six clades (α, β, γ, κ, π, and σ) and five sub-clades (γ1, γ2, γ3, γ4, and γ*) (*13*).

In this study, we assess binding of three pili: type 1, Ucl, and Yeh. Type 1 pili are tipped with the mannose binding FimH adhesin, are in the γ1 sub-clade, present in 89% of sequenced isolates, and facilitate UPEC pathogenesis in the bladder and persistence in the gut. Ucl pili are in the γ4 sub-clade and promote UPEC colonization of the mouse intestine. Yeh pili are also from the γ4 sub-clade and are found in 100% of sequenced isolates but have no known ligand or function ascribed to them (*14*–*16*). We previously found that the clinical cystitis isolate UTI89 can be depleted from the mouse intestine following oral administration of high-affinity mannose analogs that neutralize FimH function (*17*). Mannosides are in phase 1b human clinical trials for the treatment and prevention of UTI (*15*). However, the contribution of CUP pili to UPEC intestinal biogeography remains largely uncharacterized. In the present study, we discovered that adhesins from Yeh and Ucl pili bound exclusively to fecal contents in the colon lumen, while type 1 pili were unique in enabling access to the secreted mucus layers and underlying tissue. In situ and ex vivo experiments that carefully preserved the IML of the colon revealed a promiscuous binding pattern for the FimH adhesin within the mucosa, including binding to both inner and outer mucus layers, to the glycocalyx at the epithelial surface, as well as to cells within the lamina propria. Notably, a UTI89 mutant lacking functional type 1 pili up-regulated flagellar-mediated motility, which, in turn, facilitates the infiltration of UPEC into the typically impenetrable IML in an ex vivo model of bacterial-mucus interaction. Our study illustrates the role of CUP pili in establishing radial spatial organization within the gut microbiota and highlights the importance of studying compensatory regulatory mechanisms that exist between distinct colonization factors.

## RESULTS

### Luminal and mucosal binding of lectin domains from UTI89’s repertoire of CUP adhesins

**Fig. 1.**
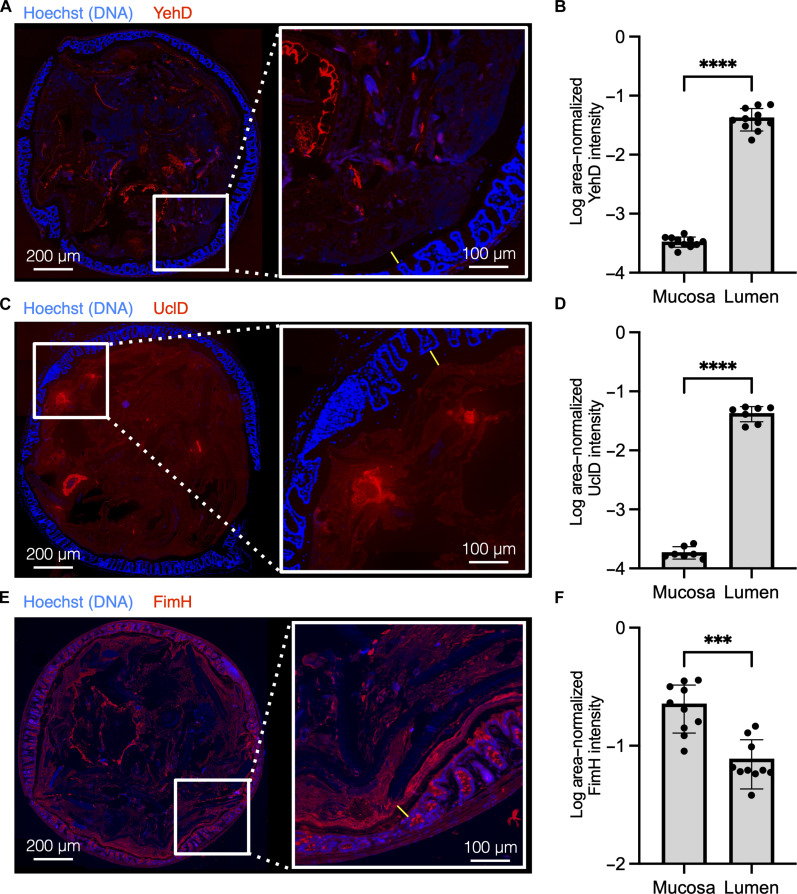
Differential binding of UTI89 CUP adhesins to colon mucosa and lumen. (**A**, **C**, and **E**) Purified lectin domains of Yeh, (YehD), F17-like (UclD), and type 1 (FimH) pili were bound to methacarn-fixed C57BL/6NJ (The Jackson Laboratory) female mouse colon sections. Lectin domain is labeled in red, and DNA is labeled in blue (Hoechst stain). On the left of (A), (C), and (E) is a representative tile scan of the entire colon section, and on the right is a magnified view of the colon mucosa. The IML is denoted by a yellow line. Left scale bars, 200 μm; right scale bars, 100 um. (**B**, **D**, and **F**) Segmentation analysis was performed on whole-colon sections to quantify the intensity of FimH binding signal in the colon mucosa and the lumen (fig. S2). [(A) and (B)] YehD binds in the colon lumen but does not bind within mucosa, *n* = 4 mice, two sections per mouse. [(C) and (D)] UclD binds within the colon lumen but does not bind within mucosa, *n* = 4 mice, two sections per mouse. (C) FimH binds both the mucosa and lumen of the colon, *n* = 5 mice, two sections per mouse.

**Fig. 2.**
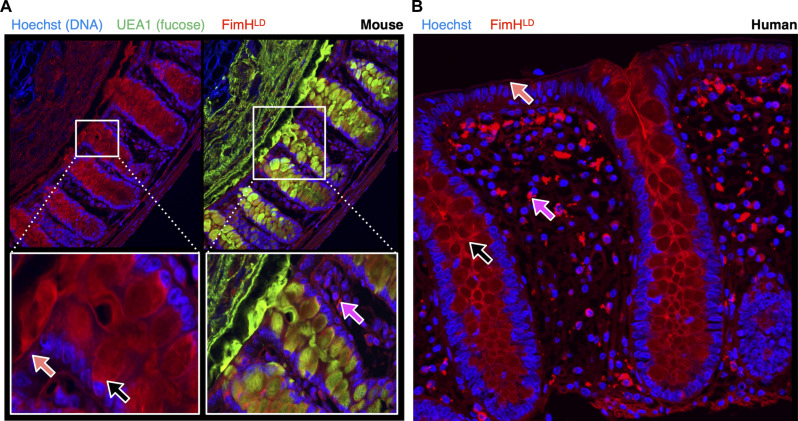
FimH binds broadly within the mouse and human colon mucosa. Fixed colon tissue was stained with FimH for high-resolution imaging of the mucosa. (**A**) C57BL/6NJ (Taconic Biosciences) female mouse distal colon was stained with FimH (red), UEA1 (green), and Hoechst (blue). IML denotes the inner mucus layer, and OML denotes the outer mucus layer, *n* = 3 mice, two sections each. (**B**) Fixed colon tissue sections from healthy human donors were stained with FimH (red) and Hoechst (blue), *n* = 3 individuals, two sections each. [(A) and (B)] Black arrows mark FimH signal on goblet cells. Orange arrows mark FimH signal on the epithelial surface. Magenta arrows mark FimH signal within the lamina propria.

We purified and fluorescently labeled the lectin domains of Yeh, F17-like, and type 1 pili (YehD, UclD, and FimH, respectively) and tested binding of these lectin domains on cross sections of the mouse distal colon, which were cut in the area where the fecal pellet was the thickest, so that the luminal contents and mucosa could be imaged simultaneously through tiled imaging of the whole-colon section. Methanol-Carnoy fixation was used to preserve the secreted mucus layers because it limits dehydration and collapse of the mucus layers (*16*). YehD appeared to bind to dietary fibers present in the intestinal lumen but did not bind the mucosa (Fig. 1, A and B). UclD also bound within the lumen, to a ligand visually distinct from the binding site of YehD, and did not bind within the mucosa (Fig. 1, C and D). Conversely, FimH bound promiscuously throughout the colon, with the strongest observed signal coming from the outer edge of colon sections, which includes the secreted mucus layers and mucosa (Figs. 1, E and F, and 2A). UclD has previously been observed to bind to epithelial tissue (*17*). Here, we found no binding of the lectin domain to colon sections. Binding differences may stem from variations in mouse strains, tissue preparation methods, or alterations in the mouse gut microbiota, which can influence host glycosylation profiles (*16*, *18*–*21*). Because glycosylation patterns can differ between mice and humans, we confirmed the negative binding of YehD and UclD colon epithelium by staining donated healthy human biopsy samples (fig. S1) (*22*, *23*).

### FimH binds to mouse and human colon mucosa in situ

Because the mucosa is a key component of host-microbe interactions, we prioritized understanding the contribution of FimH to colonization of the mouse colon. We performed high-magnification imaging of the distal colon and found that FimH binds to several areas within the mucosa. FimH stained the surface of epithelial crypts, both the inner and outer mucus layer, as well as mucus-secreting goblet cells (Fig. 2A and fig. S2B). We used the fucose-binding UEA1 lectin as a counterstain, because it is a specific marker for goblet cells and secreted mucus in mice (*24*). In addition to staining luminal components of the mucosa, FimH also stained some cells within the lamina propria. We then translated these findings to the human colon (Fig. 2B). Because these human biopsy samples were fixed in paraformaldehyde, the secreted mucus layers were not preserved before sectioning; however, we were able to recapitulate binding to the epithelial crypts, mucus-secreting goblet cells, as well as binding to the lamina propria in donated human colon sections. To confirm that the observed binding did not result from nonspecific interactions, we repeated the staining using a FimH mutant incapable of binding mannose due to a known point mutation generated in the mannose binding pocket (FimH::Q133K), which resulted in no binding (fig. S3) (*25*). Further, we demonstrated that mannoside FIM1033 prevented all binding by neutralizing FimH (fig. S4). This demonstrates that the signal from bound FimH is unlikely to result from nonspecific interactions.

### FimH binds to the secreted IML of freshly explanted mouse colon

**Fig. 3.**
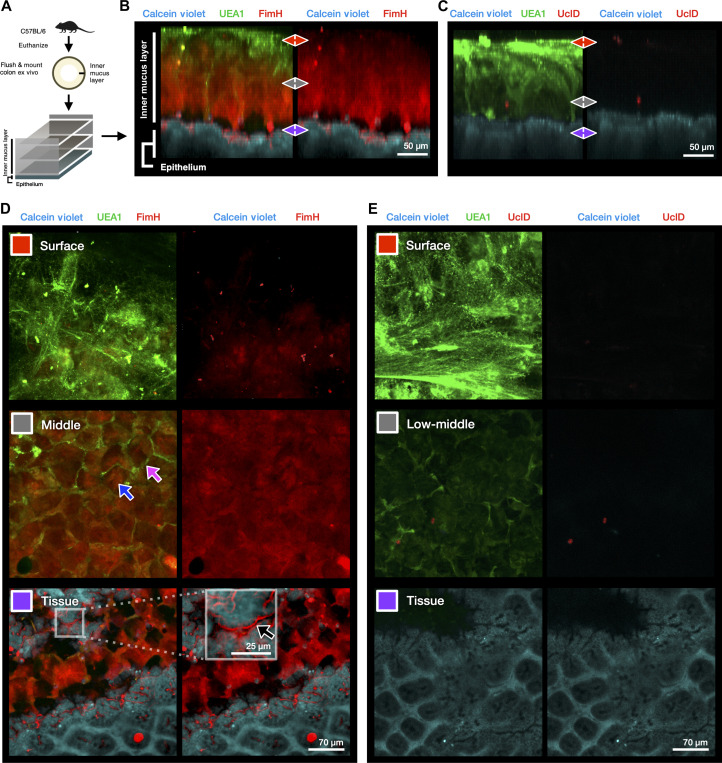
FimH binds the IML and the colon epithelial surface ex vivo. (**A**) Experimental design of 3D ex vivo mucus imaging procedure with female C57BL6/NJ (The Jackson Laboratory). (**B**) Representative side view (*x*/*z* projection) of FimH-stained reconstruction of the IML, *n* = 2 mice, and two pieces of colon per mouse. (**C**) Representative side view (*x*/*z* projection) of UclD-stained reconstruction of the IML, *n* = 4 mice, and two pieces of colon per mouse. (**D**) Selected slices (*x*/*y* projections) of three areas of interest (IML surface (red), IML middle (gray), and tissue (purple) representative of FimH-stained colon explants. Magenta arrow highlights UEA1-stained intercrypt mucus. Blue arrow highlights FimH-bound main mucus plume. Black arrow and white box inset denote FimH binding within the glycocalyx at the epithelial surface beneath the IML (fig. S5, A and B). (**E**) Selected slices (*x*/*y* projections) of IML surface (red), IML lower middle area (gray), and tissue (purple) representative of UclD-stained colon explants.

Although FimH bound to multiple regions of the colon mucosa, cross sections of fixed gut tissue necessarily expose ligands that may not be available to bacteria originating from the lumen. Mannose is prevalent in many tissues, because it serves as a building block to many N-linked glycan structures and is known to decorate many glycoproteins (*26*, *27*). To better understand the ability of FimH to bind to luminally to the colon mucosa, we used an ex vivo model in which fluorescently labeled lectins can be added apically to visualize the IML and underlying epithelium (*28*). In this model, the distal mouse colon is flushed to remove the fecal contents and outer mucus layer and subsequently mounted in a sealed imaging chamber, with the mucus facing upward (Fig. 3A). We co-stained the explanted tissue with fluorescently labeled FimH and UEA1, because UEA1 stains mucus well and specifically stains a substructure of the IML called intercrypt mucus, which is secreted by a specialized population of goblet cells at the top of colonic crypts (Fig. 3, B and D) (*24*). Unlike UEA1, FimH bound to both intercrypt mucus and to the typical plumes of secreted mucus (Fig. 3D, middle). In addition, strong binding from FimH was observed below the IML, in the glycocalyx at the surface of colonic crypts (fig. S5, A and B). The FimH::Q133K mutant, which no longer binds mannose, did not bind to secreted mucus ex vivo (fig. S5C). We also repeated the experiment using UclD as a control, and, in agreement with our binding data in Fig. 1, we did not observe binding of UclD within the IML, further confirming that mucus binding was a unique property of the FimH adhesin. To quantify the interaction of FimH with secreted mucus, we performed protein binding assays using purified FimH against commercially available bovine submaxillary mucin (BSM) (fig. S6, A and B). Here, FimH demonstrated a strong dose-dependent binding to immobilized BSM, and this binding was reversible by incubation with mannoside (fig. S6C).

### Carriage of type 1 pili promotes UTI89 association with colon mucus in vivo

**Fig. 4.**
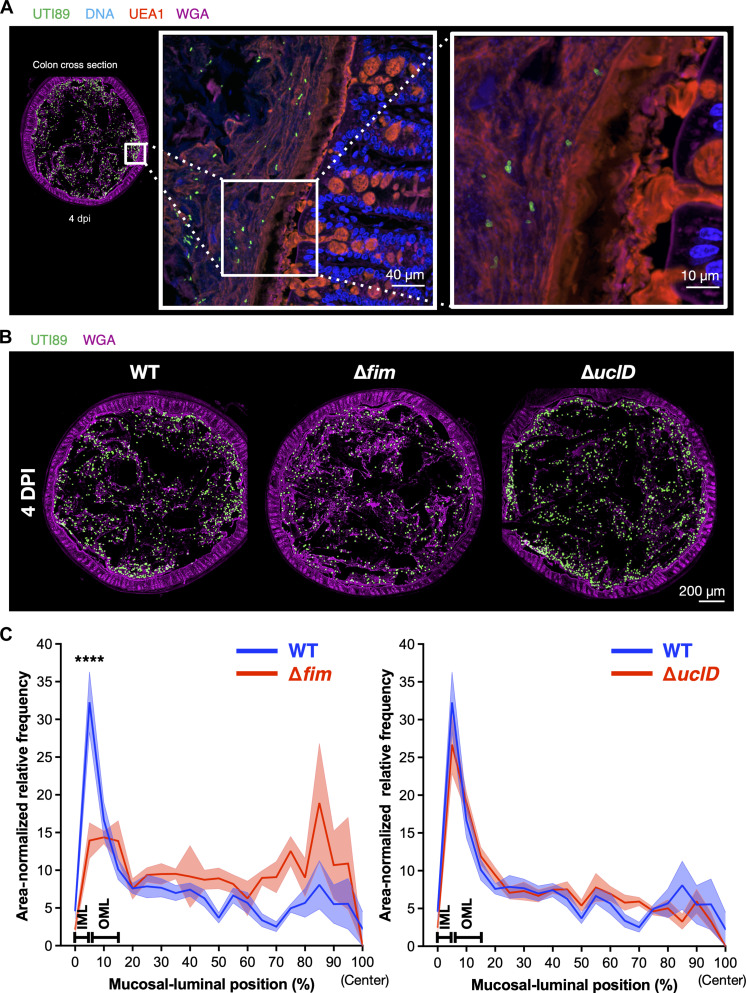
UTI89∆*fim*::kanR is defective for mucus association during colonization. Female C3H/HeN mice (Envigo) were colonized with WT, ∆*fim*, or ∆*uclD* mutant UTI89 and euthanized 4 days postinfection (dpi). Fixed colons were stained for UTI89 by using an antibody specific for the O18 lipopolysaccharide (LPS) antigen (green) and co-stained with fucose-binding UEA1 (red) and GlcNAc-binding WGA (pink) lectins to visualize mucus layers (fig. S7, A and B). DNA from epithelial cells was visualized by Hoechst stain (blue). (**A**) WT UTI89 (green) is mainly found in the lumen and outer mucus layer of the colon at 4 dpi (fig. S7C). (**B**) Representative images from tiled mouse colon sections were stained for UTI89 (green) and WGA (pink). (**C**) Quantification of radial distribution of UTI89∆*fim* and UTI89∆*uclD* (red line) compared to WT UTI89 (blue line). *n* = 5 mice per group, two sections per mouse. ****P < 0.0001.

The observation that purified FimH lectin domain can bind both secreted mucus layers as well as the surface of colonic crypts may explain why mannoside compounds are able to deplete UPEC from the mouse intestine (*17*). However, the degree to which the promiscuous in situ and ex vivo binding phenotypes of FimH contribute to mucus association of UPEC during active colonization is unknown. Because mouse intestines are intrinsically resistant to colonization by *E. coli*, we used a streptomycin pretreatment model that enables UPEC colonization in the gut to study the localization of UTI89 mutants in the colon (*17*). Mice were colonized with wild-type (WT) UTI89 or a mutant with the type 1 operon replaced by an antibiotic resistance marker (UTI89∆*fim::*kanR, subsequently referred to as UTI89∆*fim*) by oral gavage, and distal colons were harvested and fixed with methyl-Carnoy 4 days postinfection (dpi). We then localized UTI89 in situ using an antibody to the O18 lipopolysaccharide (LPS) antigen, which specifically identified UTI89 in colon sections (fig. S6, A and B). WT UTI89 was found throughout the colon lumen and between the outer mucus layer and the IML at 4 dpi, but very few cells were found within the IML (Fig. 4A and fig. S7C). When comparing the distribution of WT UTI89 and UTI89∆*fim* along the radial axis, we found UTI89∆*fim* to be significantly depleted from the mucus layers of the colon (Fig. 4, B and C). Mice were also colonized with UTI89∆*uclD* as a parallel control, and there was no difference in radial localization of the UTI89∆*uclD* mutant compared to that of the WT strain.

### UTI89∆*fim* uses flagellar-mediated motility to penetrate the mouse and human IML ex vivo

**Fig. 5.**
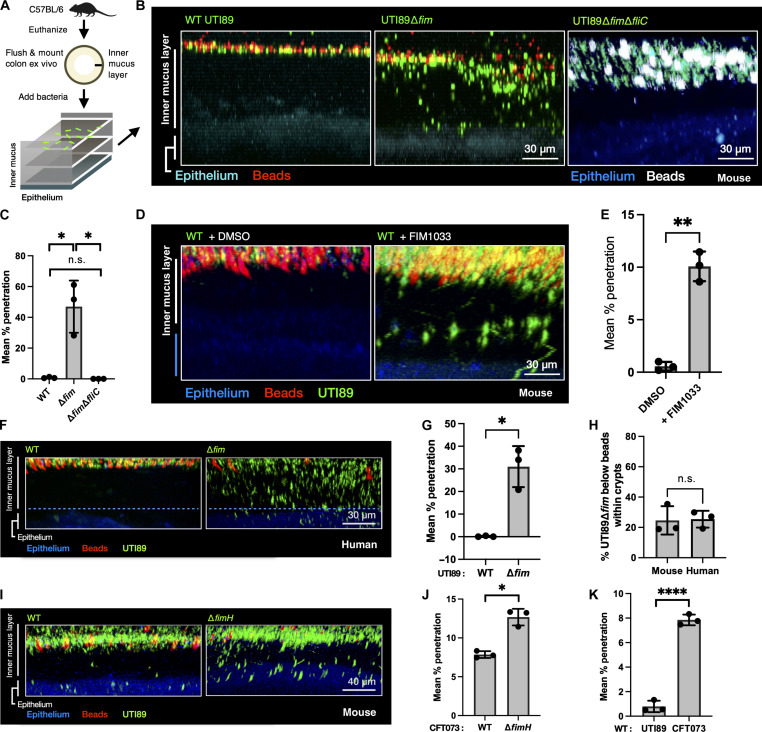
Flagella-mediated motility permits UPEC penetration of the IML ex vivo. (**A**) Experimental design of ex vivo mucus imaging procedure, with GFP-expressing UTI89 mutants added on top of freshly prepared mouse distal colon explants (female C57BL/6NJ mice, The Jackson Laboratory). Fluorescent beads (white; 1-μm size) were also added on top of each sample to determine the top of the IML. (**B**) WT UTI89 is unable to penetrate the IML, whereas UTI89∆*fim* (green) is detected below the fluorescent beads (white). Deletion of FliC allele from UTI89∆*fim* restores non-penetration phenotype. (**C**) Quantification of the proportion of each mutant penetrating below the bead-containing portion of the IML, *n* = 3 mice per group, two pieces of colon per mouse. (**D**) WT UTI89 cultures in the presence of compound FIM1033 recapitulate penetration phenotype of UTI89∆*fim*, whereas vehicle control [dimethyl sulfoxide (DMSO)] does not. (**E**) Quantification of the proportion of each group from (D), which penetrate below the bead-containing portion of the IML, *n* = 3 mice per group, two samples per colon. (**F**) UTI89∆*fim* penetrates the IML of healthy human colon explants. (**G**) Quantification of the proportion of WT UTI89 and UTI89∆*fim*, which penetrated past the bead-containing portion of the human IML, represented in (I); WT and ∆*fim*: *n* = 3 piece of colon from two individual donors. (**H**) The proportion of UTI89∆*fim* found below the bead-containing portion of the IML that is found in association with epithelial tissue is equivalent between mouse and human colons, *n* = 3 mice, two pieces per colon. (**I**) CFT073∆*fimH* penetrates the IML to a greater degree than WT CFT073. (**J**) Quantification of the proportion of WT CFT073 and CFT073∆*fimH* below the beads; WT: *n* = 3 mice, two pieces per colon; ∆*fimH*: *n* = 3 mice, two pieces per colon. (**K**) Quantitative comparison of penetration of WT UTI89 and CFT073. [(D), (G), and (I) to (K)] *P < 0.05, **P < 0.005, and ****P < 0.00005. n.s., not significant.

Although UTI89 was primarily found in the outer of the two secreted mucus layers in vivo, a small number of cells were present in or very near to the top of the IML of the colon. Having determined that FimH binds throughout the IML of fresh colon explants, we examined the behavior of UTI89 in the colon explant model using WT UTI89 and UTI89∆*fim* carrying a plasmid with constitutive expression of a green fluorescent protein (GFP) (*29*). These strains were grown in type 1 pili-inducing conditions (*30*) and added to the surface of colon explants along with 1-μm fluorescent beads, which are used to demarcate the top of the IML because the healthy IML is physically impenetrable to bacteria-sized beads or bacteria (*7*). By reconstructing the IML using confocal *z*-stacks, we showed that, while WT UTI89 did not penetrate the IML, roughly half of the UTI89∆*fim* cells infiltrated beneath the beads 15 min after being added to the explanted tissue (Fig. 5, A to C). Type 1 pili and flagella expression are inversely regulated in UTI89 (*31*). Thus, UTI89∆*fim* overexpression of flagella may explain the observed penetration phenotype. We used two-photon (2P) microscopy to acquire real-time videos of the mutants within the IML and deep within the colonic crypts. UTI89∆*fim* used active motility to dive into the colonic crypts (fig. S8, A and B, and movie S1). The necessity of flagella for mucus penetration was confirmed by testing UTI89 lacking type 1 pili as well as the major flagellar subunit FliC (UTI89∆*fim*∆*fliC*). This strain was incapable of penetrating the IML (Fig. 5, B and C). Notably, an average of 25% of the mucus-penetrating FimH mutants lodged themselves in the colon epithelium, where many remained immotile (Fig. 5H and movie S2). The penetration phenotype was also reproducible in explanted human colon tissue, where we found that UTI89∆*fim* swam through the IML and reached the epithelium in equivalent as in mice numbers (Fig. 5, F to H). We confirmed that the growth of type 1 mutants led to an up-regulation of flagellar expression (fig. S8, D and G). UPEC is an extremely genetically diverse group of *E. coli* isolates, sharing only 60% of core genetic content, exhibiting distinct phenotypes and unique transcriptional programs of shared genes even when grown in identical conditions (*32*). To determine whether the observed phenotype was specific to UTI89 or could be a shared property of UPEC, we tested whether disruption of type 1 pilus function in the clinical UPEC isolate CFT073 would enable mucus penetration through motility. CFT073∆*fimH* penetrated the IML similarly to UTI89∆*fim* (Fig. 5, I and J). Unexpectedly, WT CFT073 also penetrated the IML to a small degree, whereas WT UTI89 was never found below the beads (Fig. 5K). Similarly to UTI89, CFT073∆*fimH* penetrated the IML to a greater degree than WT CFT073 (Fig. 5J). While UPEC strains lacking type 1 pili are rare, several environmental factors have been identified that may affect regulation of type 1 pili (*33*–*35*). We simulated environmental inhibition of type 1 pili by culturing UTI89 in the presence of mannoside, which led to an up-regulation of flagellar expression and concomitant increase in the ability of wild-type UTI89 to penetrate the IML (Fig. 5, D and E, and fig. S8, C and E to G).

## DISCUSSION

The intestinal microbiota is increasingly recognized to be spatially organized, with mouse studies demonstrating how a single gene can have a major effect on the localization of colon colonizing bacteria (*36*, *37*). To date, CUP pili have been thought to promote infectious colonization by enabling attachment to host surfaces (*13*). We made the unexpected discovery that UTI89 encodes at least two CUP adhesins (YehD and UclD), which bind within the luminal or fecal contents of the mouse colon. A glycan array of UclD binding revealed affinity for more than one glycan, which may explain UclD binding in other studies (*38*).

*E. coli* grows well on mucus-derived sugars and has been isolated from secreted mucus layers of the large intestine (*10*, *39*–*44*). However, few colonization factors have been identified which permit *E. coli* to reside within mucus. The secreted mucus layers of the intestine are structured around the highly glycosylated Muc2 protein backbone (*8*). The abundant and extremely varied glycosylation patterns of the Muc2 backbone are thought to play a dual role of providing a physical barrier from bacteria while also selecting for beneficial microbes (*21*). In a healthy host, the IML of the colon restricts bacteria from accessing the underlying epithelium. However, some bacteria are able to penetrate the IML generally by expressing mucolytic enzymes (*37*, *45*), while others take advantage of disruptions of the IML during inflammatory conditions to penetrate the IML (*46*). Attachment to the top of the IML may also be a bacterial strategy to avoid peristalsis-mediated removal from the intestine (*21*).

Mannose is key building block for N-linked glycosylation across mammals and is found nearly ubiquitously throughout mammalian body sites (*27*). Terminal mannose residues, also known as high mannose, have been found in several glycoproteins present on intestinal epithelium (*47*, *48*). Colonic mucus also reportedly consists of a mannose-containing fraction (*49*). The major secreted intestinal mucin Muc2 contains 30 N-linked glycan sites, and the von Wildebrand factor domain of Muc2 is mannosylated (*50*, *51*). While the specific distribution of terminal mannose present on secreted Muc2 and glycocalyx is unclear, expression of glycosyltransferases by the microbiota can expose underlying mannose residues (*22*). Commensal *Bacteroides* species residing in the large intestine are able to access host-derived mannose through their vast repertoire of mannosidases (*52*, *53*). The degree to which these sources of mannose are exploited by UPEC in the intestine is unknown. UPEC is poised to take advantage of exposed mannose for adhesion, because successful colonization and invasion of bladder epithelium depend on type pilus-mediated binding to mannosylated glycoproteins (*54*).

Purified FimH adhesin bound throughout the entirety of colon sections, including the lumen and throughout the secreted mucus layers and underlying epithelium. On colon explants, FimH had a diffuse staining pattern in secreted mucus and bound strongly at the epithelial surface. Transmembrane mucins are embedded within the epithelial cell membrane and have a critical role in tissue repair as well as providing an additional layer of protection against bacterial invasion (*55*). Our data suggest that UPEC may interact with transmembrane mucins within the glycocalyx of the colon. In colonization studies, genetic disruption of type 1 pili led to a substantial decrease in UTI89 found within the mucus layers, supporting the notion that type 1–mediated adhesion promotes retention in the intestine through attachment to the top of the IML or within the OML. We previously demonstrated that administration of mannose-analog compounds, called mannosides, can reduce UPEC colonization in the mouse intestine (*17*). In the present study, we demonstrate that mannoside pretreatment removes the promiscuous in situ binding of FimH from the entirety of the mouse mucosa and lumen.

UTI89∆*fim* did not colonize mucus as efficiently as WT UTI89 in vivo. Intestinal mucus is turned over at a continuous and rapid pace, with mouse colon IML capable of renewing itself within 1 hour (*56*). Therefore, tight attachment to the secreted mucus layers by bacteria may not be conducive to long-term colonization of the mucosa. Nevertheless, our work demonstrates that UTI89 associates with mucus in a type 1 pilus–dependent manner.

While intestinal mucus is often degraded during intestinal inflammation, penetration of the IML by bacteria or bacteria-sized beads in healthy hosts is rare (*7*, *46*, *57*, *58*). However, we found that disruption of type 1 pili led to substantial flagellar-dependent infiltration of the normally impenetrable IML of the colon ex vivo. Flagella-mediated motility by UTI89∆*fim* and WT UTI89 grown in the presence of mannoside was sufficient for ex vivo penetration of the IML in our mouse and human colon explant experiments. These results confirm previous observation in *Salmonella* Typhimurium, demonstrating that flagella permit penetration of colon mucus (*59*). Whereas WT UTI89 did not demonstrate motility without mannoside, a small percentage of WT CFT073 *E. coli* penetrated the IML, with several of the penetrating cells being motile. UPEC has extensive genetic and functional diversity, with related UPEC isolates often displaying strong heterogeneity in transcriptional output of shared genes, even under identical culture conditions (*32*). Notably, in a study of clinical isolates, chemotaxis and motility genes are among the genes whose expression varies in similar culture conditions (*32*). Type 1 pili are under control of an invertible promoter controlling ON and OFF states of expression whose orientation is flipped by the expression of three recombinases in response to environmental signals (*60*). Down-regulation of type 1 pili expression is correlated with up-regulation of flagella (*31*). Therefore, the difference between the penetration of WT UTI89 and CFT073 may reflect a difference in regulation of the *fimS* promoter of type 1 pili between the two strains (*61*). Although WT UTI89 and CFT073 expressed similar number of flagella in type 1–inducing conditions, only CFT073 penetrated the IML. CFT073 encodes a homolog to the *pic* mucinase, which is commonly found in enteroaggregative *E. coli* (EAEC) (*62*). Pic is a serine protease that permits EAEC access to intestinal epithelium by degrading the Muc2 mucin, although this has only been described in the small intestine (*63*, *64*). UTI89 lacks mucinases, which could further explain the difference in mucus penetration between UTI89 and CFT073. Note that a third of WT UTI89 cells were flagellated, even when the bacteria displayed 0% penetration of the IML. This difference highlights the need to better understand the regulatory processes that license flagellar activity in UTI89.

Whether UPEC uses flagella to swim through the colon IML in vivo is not known. The host dedicates considerable resources to inhibit flagellar activity in the intestine, including sensing the presence of flagellar subunits and secreting antimicrobial peptides to counteract bacterial motility (*65*, *66*). Some older reports suggest that highly motile *E. coli* variants may be selected out of the mouse intestine, although these were performed using lab-adapted strains rather than clinical isolates (*67*, *68*). Recent work in the streptomycin colonization model suggests that flagella-mediated motility is required for the commensal Nissle *E. coli* strain (EcN) to make use of nitrate secreted in response to streptomycin treatment, finding that an EcN flagellar mutant has a defect in colonizing the mucosa (*36*). Traversing mucus likely represents a high-risk, high-reward program that enables access to host derived glycans and electron acceptors such as oxygen and nitrate, at the cost of alarming the immune system (*69*). Intestinal mucins and glycans can affect microbial virulence factor expression and, in some cases, have been found to induce flagellar-mediated dispersal of bacterial biofilms (*70*–*72*).

As expected, on the basis of prior observation, the IML of colonized mouse colons remained mostly devoid of colonizing UTI89 (*7*). However, our ex vivo work demonstrates the capacity of UPEC to easily penetrate the IML under conditions that down-regulate type 1 pili and up-regulate motility. That IML penetration is not seen in healthy hosts in vivo could be because it triggers host responses. In healthy hosts, the observation that flagella enable efficient penetration of the IML may explain how UPEC reside in the continuously secreted mucus layers of the colon without being washed away. Although identifying the specific signals that affect promoter inversion requires further study, our work suggests that transient flagellar expression could enable *E. coli* to forage mucin in the IML, unveiling strategies available for UPEC to maintain residence in the secreted mucus layers of the colon.

These findings highlight a potentially important balance in binding versus motility in allowing UPEC and other related strains to colonize intestinal mucus. Perhaps in inflammatory conditions, breaching the IML and directly attaching to the epithelium may be more favorable because mucus is often already degraded, feces are more fluid, and the immune system is dysregulated. Inflammation can alter host glycosylation patterns and affect virulence signaling in bacteria (*72*, *73*). Given that the rUTI intestinal microbiota shares microbial signatures with that of inflammatory bowel disease patients, future studies on the effect of intestinal inflammation on UPEC colonization factor expression may be particularly informative (*74*).

## MATERIALS AND METHODS

### Bacterial strains, plasmids, and growth conditions

Strains used in this study are presented in table S1. CUP adhesin and flagellar deletions in UTI89 were created by replacing the gene of interest with antibiotic-resistance markers using the λ Red Recombinase system, as previously described (*62*, *75*, *76*). Type 1 pilus–inducing growth conditions for UTI89 and CFT073 consist of two rounds of bacterial growth in static flasks, serially at 37°C for 24 hours, as previously described (*17*, *30*). The pcom-GFP plasmid was transformed into UTI89 and CFT073 mutants for live imaging with ex vivo colon explants, and fluorescent cultures were cultured using ampicillin (100 μg/ml) to select for the plasmid (*77*).

### Protein expression, purification, and labeling

FimH and UclD lectin domains were purified, as previously described (*17*, *78*). The lectin domain of *yehD* was cloned from UTI89, tagged with a 6-his tag, and placed into a pTRC99a expression plasmid under the control of isopropyl-β-d-thiogalactopyranoside. Protein was expressed from C600 cells, and periplasm was harvested through outer membrane digestion with lysozyme followed by cell pelleting. The supernatant containing tagged protein was purified via affinity chromatography with a cobalt resin. Lectin domains were moved to 0.1 M NaHCO_3_ (pH 8.3) buffer before fluorescent labeling and then labeled using the Thermo Fisher Scientific Alexa Fluor 647 NHS Ester (Succinimidyl Ester) kit (catalog number A20006) per the manufacturer’s recommendations.

### Lectin fluorescence studies of fixed colons

For mouse studies, 7- to 8-week-old female specific pathogen–free C57BL6/NJ mice were euthanized, and distal colon segments were fixed in methanol-Carnoy to preserve the intestinal mucus (*16*). Fixed colon samples were embedded in paraffin, and sections were cut to 5-μm thickness. Sections were then de-waxed with xylene and rehydrated using sequential incubation into 100, 95, 70, 50, and 30% EtOH. Sections were washed in phosphate-buffered saline (PBS) and then blocked in 5% fetal calf serum for 30 min at room temperature. A staining solution containing Hoechst (5 μg/ml; DNA stain) and the relevant labeled adhesin lectin domain (10 μg/ml), or Hoechst alone, was used to stain the sections for 10 min, followed by washing the slides twice in PBS. For FimH, FimH::Q133K, and YehD, the slides are dipped in 150 ml of PBS for the washing step, whereas only 250 μl of PBS was used to wash the UclD adhesin off, due to a lower binding affinity. For blocking experiment with mannoside, compound FIM1033 was incubated at 10× molar excess with FimH in PBS 0.05% Tween 20 for 30 min at room temperature minutes before continuing with the staining protocol as above. FIM1033 is referred to as 29R in a previous publication (*79*). For human colon sections, sigmoid colon biopsies representative of three healthy individuals (two males and one female), aged 56 to 70 years old, undergoing routine endoscopy screening were collected at Sahlgrenska University Hospital (Gothenburg, Sweden) and processed, as previously described (*57*). For high-resolution imaging of FimH stains on mouse and human colon sections (Fig. 2), female C57BL6/NJ mice from Taconic Biosciences were used, and experiments were performed in the Department of Medical Biochemistry and Cell Biology at the Institute of Biomedicine, University of Gothenburg in Gothenburg, Sweden, and imaged using a Zeiss LSM700 confocal microscope with a 20× air objective lens. Whole-colon tiled scans of FimH, UclD, and YehD binding, FimH::Q133K binding, and mannoside blocking experiments were performed on C57BL/6NJ mice from The Jackson Laboratory in the Department of Molecular Microbiology, Washington University School of Medicine, in Saint Louis, Missouri, using the confocal function of a Zeiss Cell Observer Spinning Disk Confocal Microscope with a 10× objective lens. Segmentation analysis was used to compare the relative intensity of luminal versus mucosal lectin binding signal. The surfaces tool of Imaris 9 software was used to isolate the mucosa (lamina propria, epithelium, and IML) from the colon muscle layer and the lumen contents (fecal matter and/or outer mucus layer) (see fig. S2C). Measurements of surface area and total intensity of the mucosa and lumen were measured in Imaris 9 and used to calculate the area-normalized intensity for each section.

### In vitro protein binding studies

Purified FimH, FimH::Q133K, YehD, and UclD proteins were placed in 1× PBS for biotinylation. Individual proteins were biotinylated with a ratio of 20:1 biotin to protein. Biotinylation occurred at 4°C with agitation for 2 hours, and proteins were subsequently dialyzed into either 1× PBS or 20 mM MES and 5.79 and 50 mM NaCl. BSM (Alfa Aesar) was diluted to 10 mg/ml, and 100 μl was used to coat to each well of Immulon 4HBX plates (Thermo Fisher Scientific, 3855). The wells were then blocked for ≥4 hours to overnight at 4°C with a blocking buffer of 1× PBS and 1%. Protein was added to the top row at 20 μg/ml, and seven serial twofold dilutions were performed on the rest of the plate. The plate was then allowed to incubate with adhered ligand overnight at 4°C diluted in a blocking buffer. Wells were then washed three times with wash buffer of 1× PBS and 0.05% Tween 20. Streptavidin–horseradish peroxidase was added at 1:1000 dilution in a blocking buffer and incubated for ~1 hour at 4°C. Wells were washed two times with wash buffer and then one time with 1× PBS. Wells were developed with 1:1 tetramethylbenzidine development reagent and quenched with ~1 M H_2_SO_4_. Wells were read at 450 nm. For mannoside inhibition assays, proteins were added at 5 μg/ml in all wells of the plate, and mannoside FIM-1033 was added to top row at 1 μg/ml, and serial twofold dilutions were performed to complete the dilution series.

### Lectin fluorescence studies of ex vivo colon

Colon explants of specific pathogen–free 7- to 8-week-old female C57BL6/NJ mice from Taconic Biosciences or female C57BL6/NJ mice from The Jackson Laboratory were prepared, as previously described, for the preservation of the IML (*24*, *28*). Briefly, distal colons were flushed with Krebs buffer to remove luminal content and unattached mucus. The muscle layer was removed by microdissection, and the distal colon was mounted in a horizontal chamber system and maintained with basolateral Krebs-glucose buffer and apical Krebs-mannitol buffer. Calcein violet was used with Krebs-glucose buffer to label epithelial tissue. Fluorescent beads (1 μm) were used to verify the penetrability properties of the IML, ensuring intact mucus layers. Rhodamine-labeled UEA1 lectin and Alexa Fluor 647–labeled FimH were added on top of mucus preparations at 50 μg/ml to label the IML. *Z*-stacks were acquired using LSM700 with a Plan-Apochromat 20×/1.0 differential interference contrast water objective (Zeiss) and the ZEN 2010 software. Imaging of the FimH:Q133K mutant was performed on C57BL/6NJ mice from The Jackson Laboratory in the Department of Molecular Microbiology, Washington University School of Medicine, in Saint Louis, Missouri.

### Immunohistochemistry of UTI89 in colon sections

Specific pathogen–free 7- to 8-week-old female C3H/HeN mice from Envigo Laboratories were colonized in the streptomycin model as described above and euthanized at 4 dpi along with noninfected controls. Colons were freshly harvested, fixed in methanol-Carnoy, and processed as for lectin fluorescence studies, as previously described (*16*). Antigen retrieval was performed at near-boiling temperature using citrate buffer. A primary antibody specific to *E. coli* O18 LPS was used at a dilution of 1:500 in 5% fetal bovine serum to probe for UTI89, and a secondary antibody conjugated to Alexa Fluor 488 was used to detect the primary. Tile scans acquired using LSM700 20× air objective lens (8 × 8 tiles, 1024 × 1024 pixel resolution, averaging two layers).

### Quantification of UTI89 biogeography

The epithelial surface of stained mouse colon sections was manually mapped using Bitplane Imaris Software, and O18-positive cells were mapped using the Imaris “surfaces” function. *X*/*Y* coordinate data were extracted from each colon section, with the sample center defined as the intersection of the minimum/maximum *X*/*Y* axis lines. O18-positive cell counts were calculated along the center-mucus radial line (scaled from 0 to 100% and binned into 5% groups). Absolute cell counts were divided by relative bin area to account for the fact that the sampling area for each bin decreases along the mucus-center radial axis. Quantification of bacteria in the IML was done by manually identifying bacteria present within IML structures stained by wheat germ agglutinin (WGA) and unstained by Hoechst.

### Ex vivo bacterial penetration assay and imaging

Colon explants of naive 7- to 8-week-old female C57BL6/NJ mice were prepared preserve to structure of the IML, as previously described. For human colon studies, freshly isolated sections of human colon were obtained at ileocolonic resection surgery from the Digestive Diseases Research Core Center at Washington University. UTI89 mutants expressing the pCom-GFP plasmid were grown in type 1–inducing conditions. UTI89 mutants were resuspended to an optical density at 600 nm (OD_600_) of 0.6 in PBS, mixed with 1-μm-sized FluoSpheres before being added on top of colon preparations for 15 min, and imaged. UTI89 bacterial mutants were first imaged using an LSM700 confocal microscope and subsequently at a higher rate of capture by 2P microscopy. 2P imaging was performed with a custom-built dual-laser 2P microscope equipped with a 1.0 numerical aperture 20× water dipping objective (Olympus). Samples were excited with a Chameleon Vision II Ti:Sapphire laser (Coherent) tuned from 750 to 980 nm depending on the experiment and fluorescence emission detected by photomultiplier tubes simultaneously using appropriate emission filters to separate second-harmonic generation and the various fluorescence signals. Three-dimensional (3D) images were collected (61 *z* steps, 400 μm by 400 μm by 60μm) from the luminal side to assess bacterial penetration of the mucus and attachment to the epithelium, as previously described (*80*, *81*). For human tissue imaging, deidentified colon biopsy specimens were obtained through the Wash U Digestive Diseases Research Core Center. Tissue was placed in oxygenated Gibco CO_2_-independent medium at 37°C, otherwise treated similarly to mouse colon tissue, with imaging performed within 2 hours of tissue acquisition. For assessment of bacterial motility, 2P imaging was used to capture time-lapse video images of GFP-expressing mutants and FluoSpheres within mucus samples. Bacterial cells and beads were tracked in Imaris 9 software, and motility was assessed using celltrackR/MotilityLab (2Ptrack.net).

### Quantification of *E. coli* mucus penetration

UTI89 and CFT073 penetration of the IML was measured on 3D *z*-stacks captured using 2P microscopy of treated colon explants (as described above). 3D images were manually segmented on the basis of regions of interest. Fluorescent beads (1 μm) were used to define the “top” of the IML. The spot function of Imaris 9 software was used to identify individual bacteria, and the elongation of the point-spread function was modeled using default settings (5-μm estimated *XY* diameter, and 11.7-μm estimated *Z* diameter). The number of spots was gated using spot quality, using size and brightness estimates of each spot. Each image was manually reviewed, and glaring errors in identified spots, such as missing spots or extraneous spots, were manually corrected. To calculate the degree of penetration, the number of GFP-positive cells below the bead-containing top of the mucus layer was divided by the total number of bacteria. To calculate the number of mucus-penetrative *E. coli* that entered the epithelial tissue, the number of bacteria in the region containing epithelial tissue was divided by the number of bacteria below the top of the mucus layer (below the beads). Spot counts from three individual biological replicates of each mutant were comparable across experiments, so the percentage of penetration for each mutant was averaged across all experiments from the same mutant.

### Transmission electron microscopy

For analyses by electron microscopy, bacterial cultures at OD_600_ of 0.6 were allowed to absorb onto freshly glow discharged formvar/carbon-coated copper grids (200 mesh, Ted Pella Inc., Redding, CA) for 10 min. Grids were then washed two times in dH_2_O and stained with 1% aqueous uranyl acetate (Ted Pella Inc.) for 1 min. Excess liquid was gently wicked off, and grids were allowed to air dry. Samples were viewed on a JEOL 1200EX transmission electron microscope (JEOL USA, Peabody, MA) equipped with an AMT 8 megapixel digital camera (Advanced Microscopy Techniques, Woburn, MA).

### Statistical analysis

Statistical comparison of mucosal versus luminal signal intensities from stained colon sections was performed using a paired *t* test for each lectin domain. ***P < 0.0005 and ****P < 0.00005. To determine significance of radial distribution of bacterial mutants of immunohistochemistry colon stains, the absolute cell counts from each binned decile of the colon surface area was analyzed by two-way analysis of variance (ANOVA), and Fisher’s least significant difference test was used to generate a *P* value for each bin. ****P < 0.0001. To determine statistical significance of bacterial penetration in the ex vivo mucus model, significance was determined by performing Mann-Whitney *U* test on percentages of bacteria that penetrated below the level of 1-μm beads. When a piece of colon was split to test two bacterial mutants separately, a paired *t* test was performed.

## References

[1] T. Mazzulli, Resistance trends in urinary tract pathogens and impact on management. J. Urol. 168, 1720–1722 (2002). 12352343 10.1097/01.ju.0000028385.10311.c9

[2] A. V. Franco, Recurrent urinary tract infections. Best Pract. Res. Clin. Obstet. Gynaecol. 19, 861–873 (2005). 16298166 10.1016/j.bpobgyn.2005.08.003

[3] B. Foxman, Urinary tract infection syndromes: occurrence, recurrence, bacteriology, risk factors, and disease burden. Infect. Dis. Clin. North Am. 28, 1–13 (2014). 24484571 10.1016/j.idc.2013.09.003

[4] N. Maddali, A. Cantin, S. Koshy, E. Eiting, M. Fedorenko, Antibiotic prescribing patterns for adult urinary tract infections within emergency department and urgent care settings. Am. J. Emerg. Med. 45, 464–471 (2021). 33067064 10.1016/j.ajem.2020.09.061

[5] C. W. Seymour, F. Gesten, H. C. Prescott, M. E. Friedrich, T. J. Iwashyna, G. S. Phillips, S. Lemeshow, T. Osborn, K. M. Terry, M. M. Levy, Time to treatment and mortality during mandated emergency care for sepsis. N. Engl. J. Med. 376, 2235–2244 (2017). 28528569 10.1056/NEJMoa1703058 PMC5538258

[6] D. Scholes, T. M. Hooton, P. L. Roberts, A. E. Stapleton, K. Gupta, W. E. Stamm, Risk factors for recurrent urinary tract infection in young women. J. Infect. Dis. 182, 1177–1182 (2000). 10979915 10.1086/315827

[7] G. C. Hansson, M. E. Johansson, The inner of the two Muc2 mucin-dependent mucus layers in colon is devoid of bacteria. Gut. Microbes 1, 51–54 (2010). 21327117 10.4161/gmic.1.1.10470 PMC3035142

[8] M. E. Johansson, J. M. Larsson, G. C. Hansson, The two mucus layers of colon are organized by the MUC2 mucin, whereas the outer layer is a legislator of host-microbial interactions. Proc. Natl. Acad. Sci. U.S.A. 108, 4659–4665 (2011). 20615996 10.1073/pnas.1006451107 PMC3063600

[9] M. E. Johansson, G. C. Hansson, Immunological aspects of intestinal mucus and mucins. Nat. Rev. Immunol. 16, 639–649 (2016). 27498766 10.1038/nri.2016.88 PMC6435297

[10] L. K. Poulsen, F. Lan, C. S. Kristensen, P. Hobolth, S. Molin, K. A. Krogfelt, Spatial distribution of Escherichia coli in the mouse large intestine inferred from rRNA in situ hybridization. Infect. Immun. 62, 5191–5194 (1994). 7927805 10.1128/iai.62.11.5191-5194.1994 PMC303247

[11] O. Tenaillon, D. Skurnik, B. Picard, E. Denamur, The population genetics of commensal *Escherichia coli*. Nat. Rev. Microbiol. 8, 207–217 (2010). 20157339 10.1038/nrmicro2298

[12] J. Martindale, D. Stroud, E. R. Moxon, C. M. Tang, Genetic analysis of *Escherichia coli* K1 gastrointestinal colonization. Mol. Biol. 37, 1293–1305 (2000). 10.1046/j.1365-2958.2000.02088.x 10998163

[13] D. J. Wurpel, S. A. Beatson, M. Totsika, N. K. Petty, M. A. Schembri, Chaperone-usher fimbriae of *Escherichia coli*. PLOS ONE 8, e52835 (2013). 23382825 10.1371/journal.pone.0052835 PMC3559732

[14] C. N. Spaulding, S. J. Hultgren, Adhesive pili in UTI pathogenesis and drug development. Pathogens 5, 30 (2016). 26999218 10.3390/pathogens5010030 PMC4810151

[15] GlaxoSmithKline, “NCT05138822, Safety, Tolerability, pharmacokinetic and microbiological investigation of GSK3882347 in female participants with urinary tract infections” (2024). https://clinicaltrials.gov/.

[16] M. E. Johansson, G. C. Hansson, Preservation of mucus in histological sections, immunostaining of mucins in fixed tissue, and localization of bacteria with FISH. Methods Mol. Biol. 842, 229–235 (2012). 22259139 10.1007/978-1-61779-513-8_13

[17] C. N. Spaulding, R. D. Klein, S. Ruer, A. L. Kau, H. L. Schreiber, Z. T. Cusumano, K. W. Dodson, J. S. Pinkner, D. H. Fremont, J. W. Janetka, H. Remaut, J. I. Gordon, S. J. Hultgren, Selective depletion of uropathogenic *E. coli* from the gut by a FimH antagonist. Nature 546, 528–532 (2017). 28614296 10.1038/nature22972 PMC5654549

[18] A. M. Rodriguez-Pineiro, M. E. Johansson, The colonic mucus protection depends on the microbiota. Gut Microbes 6, 326–330 (2015). 26305453 10.1080/19490976.2015.1086057 PMC4826096

[19] M. E. Johansson, H. E. Jakobsson, J. Holmen-Larsson, A. Schutte, A. Ermund, A. M. Rodriguez-Pineiro, L. Arike, C. Wising, F. Svensson, F. Backhed, G. C. Hansson, Normalization of host intestinal mucus layers requires long-term microbial colonization. Cell Host Microbe 18, 582–592 (2015). 26526499 10.1016/j.chom.2015.10.007 PMC4648652

[20] S. L. La Rosa, M. P. Ostrowski, A. Vera-Ponce de Leon, L. S. McKee, J. Larsbrink, V. G. Eijsink, E. C. Lowe, E. C. Martens, P. B. Pope, Glycan processing in gut microbiomes. Curr. Opin. Microbiol. 67, 102143 (2022). 35338908 10.1016/j.mib.2022.102143

[21] A. S. Luis, G. C. Hansson, Intestinal mucus and their glycans: A habitat for thriving microbiota. Cell Host Microbe 31, 1087–1100 (2023). 37442097 10.1016/j.chom.2023.05.026 PMC10348403

[22] L. Arike, J. Holmen-Larsson, G. C. Hansson, Intestinal Muc2 mucin O-glycosylation is affected by microbiota and regulated by differential expression of glycosyltranferases. Glycobiology 27, 318–328 (2017). 28122822 10.1093/glycob/cww134 PMC5444243

[23] J. M. Holmen Larsson, K. A. Thomsson, A. M. Rodriguez-Pineiro, H. Karlsson, G. C. Hansson, Studies of mucus in mouse stomach, small intestine, and colon. III. Gastrointestinal Muc5ac and Muc2 mucin O-glycan patterns reveal a regiospecific distribution. Am. J. Physiol. Gastrointest. Liver Physiol. 305, G357–G363 (2013). 23832516 10.1152/ajpgi.00048.2013 PMC3761246

[24] E. E. L. Nyström, B. Martinez-Abad, L. Arike, G. M. H. Birchenough, E. B. Nonnecke, P. A. Castillo, F. Svensson, C. L. Bevins, G. C. Hansson, M. E. V. Johansson, An intercrypt subpopulation of goblet cells is essential for colonic mucus barrier function. Science 372, eabb1590 (2021). 33859001 10.1126/science.abb1590 PMC8542866

[25] C. S. Hung, J. Bouckaert, D. Hung, J. Pinkner, C. Widberg, A. DeFusco, C. G. Auguste, R. Strouse, S. Langermann, G. Waksman, S. J. Hultgren, Structural basis of tropism of Escherichia coli to the bladder during urinary tract infection. Mol. Microbiol. 44, 903–915 (2002). 12010488 10.1046/j.1365-2958.2002.02915.x

[26] H. H. Freeze, Understanding human glycosylation disorders: biochemistry leads the charge. J. Biol. Chem. 288, 6936–6945 (2013). 23329837 10.1074/jbc.R112.429274 PMC3591604

[27] I. Loke, D. Kolarich, N. H. Packer, M. Thaysen-Andersen, Emerging roles of protein mannosylation in inflammation and infection. Mol. Aspects Med. 51, 31–55 (2016). 27086127 10.1016/j.mam.2016.04.004

[28] J. K. Gustafsson, A. Ermund, M. E. Johansson, A. Schutte, G. C. Hansson, H. Sjovall, An ex vivo method for studying mucus formation, properties, and thickness in human colonic biopsies and mouse small and large intestinal explants. Am. J. Physiol. Gastrointest. Liver Physiol. 302, G430–G438 (2012). 22159279 10.1152/ajpgi.00405.2011 PMC4073982

[29] K. J. Wright, P. C. Seed, S. J. Hultgren, Uropathogenic *Escherichia coli* flagella aid in efficient urinary tract colonization. Infect. Immun. 73, 7657–7668 (2005). 16239570 10.1128/IAI.73.11.7657-7668.2005 PMC1273872

[30] S. J. Hultgren, T. N. Porter, A. J. Schaeffer, J. L. Duncan, Role of type 1 pili and effects of phase variation on lower urinary tract infections produced by Escherichia coli. Infect. Immun. 50, 370–377 (1985). 2865209 10.1128/iai.50.2.370-377.1985 PMC261959

[31] S. E. Greene, J. S. Pinkner, E. Chorell, K. W. Dodson, C. L. Shaffer, M. S. Conover, J. Livny, M. Hadjifrangiskou, F. Almqvist, S. J. Hultgren, Pilicide ec240 disrupts virulence circuits in uropathogenic *Escherichia coli*. mBio 5, e02038 (2014). 25352623 10.1128/mBio.02038-14 PMC4217179

[32] H. L. Schreiber IV, M. S. Conover, W.-C. Chou, M. E. Hibbing, A. L. Manson, K. W. Dodson, T. J. Hannan, P. L. Roberts, A. E. Stapleton, T. M. Hooton, J. Livny, A. M. Earl, S. J. Hultgren, Bacterial virulence phenotypes of *Escherichia coli* and host susceptibility determine risk for urinary tract infections. Sci. Transl. Med. 9, eaaf1283 (2017). 28330863 10.1126/scitranslmed.aaf1283 PMC5653229

[33] S. El-Labany, B. K. Sohanpal, M. Lahooti, R. Akerman, I. C. Blomfield, Distant cis-active sequences and sialic acid control the expression of fimB in *Escherichia coli* K-12. Mol. Microbiol. 49, 1109–1118 (2003). 12890032 10.1046/j.1365-2958.2003.03624.x

[34] B. K. Sohanpal, S. El-labany, M. Lahooti, J. A. Plumbridge, I. C. Blomfield, Integrated regulatory responses of *fimB* to *N*-acetylneuraminic (sialic) acid and GlcNAc in *Escherichia coli* K-12. Proc. Natl. Acad. Sci. U.S.A. 101, 16322–16327 (2004). 15534208 10.1073/pnas.0405821101 PMC526197

[35] B. K. Sohanpal, S. Friar, J. Roobol, J. A. Plumbridge, I. C. Blomfield, Multiple co-regulatory elements and IHF are necessary for the control of *fimB* expression in response to sialic acid and *N*-acetylglucosamine in *Escherichia coli* K-12. Mol. Microbiol. 63, 1223–1236 (2007). 17238917 10.1111/j.1365-2958.2006.05583.x

[36] M. J. Liou, B. M. Miller, Y. Litvak, H. Nguyen, D. E. Natwick, H. P. Savage, J. A. Rixon, S. P. Mahan, H. Hiyoshi, A. W. L. Rogers, E. M. Velazquez, B. P. Butler, S. R. Collins, S. J. Mc Sorley, R. M. Harshey, M. X. Byndloss, S. I. Simon, A. J. Bäumler, Host cells subdivide nutrient niches into discrete biogeographical microhabitats for gut microbes. Cell Host Microbe 30, 836–847.e6 (2022). 35568027 10.1016/j.chom.2022.04.012 PMC9187619

[37] G. P. Donaldson, S. M. Lee, S. K. Mazmanian, Gut biogeography of the bacterial microbiota. Nat. Rev. Microbiol. 14, 20–32 (2016). 26499895 10.1038/nrmicro3552 PMC4837114

[38] S. J. Hancock, A. W. Lo, T. Ve, C. J. Day, L. Tan, A. A. Mendez, M. D. Phan, N. T. K. Nhu, K. M. Peters, A. C. Richards, B. A. Fleming, C. Chang, D. H. Y. Ngu, B. M. Forde, T. Haselhorst, K. G. K. Goh, S. A. Beatson, M. P. Jennings, M. A. Mulvey, B. Kobe, M. A. Schembri, Ucl fimbriae regulation and glycan receptor specificity contribute to gut colonisation by extra-intestinal pathogenic Escherichia coli. PLOS Pathog. 18, e1010582 (2022). 35700218 10.1371/journal.ppat.1010582 PMC9236248

[39] P. S. Cohen, R. Rossoll, V. J. Cabelli, S. L. Yang, D. C. Laux, Relationship between the mouse colonizing ability of a human fecal Escherichia coli strain and its ability to bind a specific mouse colonic mucous gel protein. Infect. Immun. 40, 62–69 (1983). 6339411 10.1128/iai.40.1.62-69.1983 PMC264818

[40] E. A. Wadolkowski, D. C. Laux, P. S. Cohen, Colonization of the streptomycin-treated mouse large intestine by a human fecal Escherichia coli strain: Role of growth in mucus. Infect. Immun. 56, 1030–1035 (1988). 3281898 10.1128/iai.56.5.1030-1035.1988 PMC259757

[41] J. V. Newman, R. Kolter, D. C. Laux, P. S. Cohen, Role of leuX in Escherichia coli colonization of the streptomycin-treated mouse large intestine. Microb. Pathog. 17, 301–311 (1994). 7723657 10.1006/mpat.1994.1076

[42] N. J. Sweeney, P. Klemm, B. A. McCormick, E. Moller-Nielsen, M. Utley, M. A. Schembri, D. C. Laux, P. S. Cohen, The Escherichia coli K-12 gntP gene allows E. coli F-18 to occupy a distinct nutritional niche in the streptomycin-treated mouse large intestine. Infect. Immun. 64, 3497–3503 (1996). 8751890 10.1128/iai.64.9.3497-3503.1996 PMC174254

[43] T. R. Licht, T. Tolker-Nielsen, K. Holmstrom, K. A. Krogfelt, S. Molin, Inhibition of *Escherichia coli* precursor-16S rRNA processing by mouse intestinal contents. Environ. Microbiol. 1, 23–32 (1999). 11207715 10.1046/j.1462-2920.1999.00001.x

[44] D.-E. Chang, D. J. Smalley, D. L. Tucker, M. P. Leatham, W. E. Norris, S. J. Stevenson, A. B. Anderson, J. E. Grissom, D. C. Laux, P. S. Cohen, T. Conway, Carbon nutrition of *Escherichia coli* in the mouse intestine. Proc. Natl. Acad. Sci. U.S.A. 101, 7427–7432 (2004). 15123798 10.1073/pnas.0307888101 PMC409935

[45] G. P. Donaldson, W. C. Chou, A. L. Manson, P. Rogov, T. Abeel, J. Bochicchio, D. Ciulla, A. Melnikov, P. B. Ernst, H. Chu, G. Giannoukos, A. M. Earl, S. K. Mazmanian, Spatially distinct physiology of *Bacteroides fragilis* within the proximal colon of gnotobiotic mice. Nat. Microbiol. 5, 746–756 (2020). 32152589 10.1038/s41564-020-0683-3 PMC7426998

[46] M. E. Johansson, J. K. Gustafsson, J. Holmen-Larsson, K. S. Jabbar, L. Xia, H. Xu, F. K. Ghishan, F. A. Carvalho, A. T. Gewirtz, H. Sjovall, G. C. Hansson, Bacteria penetrate the normally impenetrable inner colon mucus layer in both murine colitis models and patients with ulcerative colitis. Gut 63, 281–291 (2014). 23426893 10.1136/gutjnl-2012-303207 PMC3740207

[47] J. J. L. Ho, R. S. Jaituni, S. C. Crawley, S. C. Yang, J. R. Gum, Y. S. Kim, N-glycosylation is required for the surface localization of MUC17 mucin. Int. J. Oncol. 23, 585–592 (2003). 12888891

[48] K. Hase, K. Kawano, T. Nochi, G. S. Pontes, S. Fukuda, M. Ebisawa, K. Kadokura, T. Tobe, Y. Fujimura, S. Kawano, A. Yabashi, S. Waguri, G. Nakato, S. Kimura, T. Murakami, M. Iimura, K. Hamura, S. Fukuoka, A. W. Lowe, K. Itoh, H. Kiyono, H. Ohno, Uptake through glycoprotein 2 of FimH^+^ bacteria by M cells initiates mucosal immune response. Nature 462, 226–230 (2009). 19907495 10.1038/nature08529

[49] R. H. Teague, D. Fraser, J. R. Clamp, Changes in monosaccharide content of mucous glycoproteins in ulcerative colitis. Br. Med. J. 2, 645–646 (1973). 4714849 10.1136/bmj.2.5867.645 PMC1589679

[50] N. Asker, M. A. Axelsson, S. O. Olofsson, G. C. Hansson, Dimerization of the human MUC2 mucin in the endoplasmic reticulum is followed by a N-glycosylation-dependent transfer of the mono- and dimers to the Golgi apparatus. J. Biol. Chem. 273, 18857–18863 (1998). 9668061 10.1074/jbc.273.30.18857

[51] C. Werlang, G. Cárcarmo-Oyarce, K. Ribbeck, Engineering mucus to study and influence the microbiome. Nat. Rev. Mater. 4, 134–145 (2019). 40084234 10.1038/s41578-018-0079-7 PMC11906034

[52] B. Trastoy, J. J. Du, E. H. Klontz, C. Li, J. O. Cifuente, L.-X. Wang, E. J. Sundberg, M. E. Guerin, Structural basis of mammalian high-mannose N-glycan processing by human gut Bacteroides. Nat. Commun. 11, 899 (2020). 32060313 10.1038/s41467-020-14754-7 PMC7021837

[53] J. L. Sonnenburg, C. T. Chen, J. I. Gordon, Genomic and metabolic studies of the impact of probiotics on a model gut symbiont and host. PLOS Biol. 4, e413 (2006). 17132046 10.1371/journal.pbio.0040413 PMC1661682

[54] J. J. Martinez, M. A. Mulvey, J. D. Schilling, J. S. Pinkner, S. J. Hultgren, Type 1 pilus-mediated bacterial invasion of bladder epithelial cells. EMBO J. 19, 2803–2812 (2000). 10856226 10.1093/emboj/19.12.2803 PMC203355

[55] J. P. M. van Putten, K. Strijbis, Transmembrane mucins: Signaling receptors at the intersection of inflammation and cancer. J. Innate Immun. 9, 281–299 (2017). 28052300 10.1159/000453594 PMC5516414

[56] M. E. Johansson, Fast renewal of the distal colonic mucus layers by the surface goblet cells as measured by in vivo labeling of mucin glycoproteins. PLOS ONE 7, e41009 (2012). 22815896 10.1371/journal.pone.0041009 PMC3398881

[57] S. van der Post, K. S. Jabbar, G. Birchenough, L. Arike, N. Akhtar, H. Sjovall, M. E. V. Johansson, G. C. Hansson, Structural weakening of the colonic mucus barrier is an early event in ulcerative colitis pathogenesis. Gut 68, 2142–2151 (2019). 30914450 10.1136/gutjnl-2018-317571 PMC6872445

[58] S. Danese, C. Fiocchi, Ulcerative colitis. N. Engl. J. Med. 365, 1713–1725 (2011). 22047562 10.1056/NEJMra1102942

[59] M. Furter, M. E. Sellin, G. C. Hansson, W. D. Hardt, Mucus architecture and near-surface swimming affect distinct *Salmonella* Typhimurium infection patterns along the murine intestinal tract. Cell Rep. 27, 2665–2678.e3 (2019). 31141690 10.1016/j.celrep.2019.04.106 PMC6547020

[60] J. M. Abraham, C. S. Freitag, J. R. Clements, B. I. Eisenstein, An invertible element of DNA controls phase variation of type 1 fimbriae of Escherichia coli. Proc. Natl. Acad. Sci. U.S.A. 82, 5724–5727 (1985). 2863818 10.1073/pnas.82.17.5724 PMC390624

[61] M. Hung, E. Chang, R. Hussein, K. Frazier, J. E. Shin, S. Sagawa, H. N. Lim, Modulating the frequency and bias of stochastic switching to control phenotypic variation. Nat. Commun. 5, 4574 (2014). 25087841 10.1038/ncomms5574

[62] R. A. Welch, V. Burland, G. Plunkett III, P. Redford, P. Roesch, D. Rasko, E. L. Buckles, S. R. Liou, A. Boutin, J. Hackett, D. Stroud, G. F. Mayhew, D. J. Rose, S. Zhou, D. C. Schwartz, N. T. Perna, H. L. Mobley, M. S. Donnenberg, F. R. Blattner, Extensive mosaic structure revealed by the complete genome sequence of uropathogenic *Escherichia coli*. Proc. Natl. Acad. Sci. U.S.A. 99, 17020–17024 (2002). 12471157 10.1073/pnas.252529799 PMC139262

[63] A. Sheikh, T. Wangdi, T. J. Vickers, B. Aaron, M. Palmer, M. J. Miller, S. Kim, C. Herring, R. Simoes, J. A. Crainic, J. C. Gildersleeve, S. van der Post, G. C. Hansson, J. M. Fleckenstein, Enterotoxigenic *Escherichia coli* degrades the host MUC2 mucin barrier to facilitate critical pathogen-enterocyte interactions in human small intestine. Infect. Immun. 90, e0057221 (2022). 34807735 10.1128/iai.00572-21 PMC8853678

[64] P. Kumar, Q. Luo, T. J. Vickers, A. Sheikh, W. G. Lewis, J. M. Fleckenstein, EatA, an immunogenic protective antigen of enterotoxigenic *Escherichia coli*, degrades intestinal mucin. Infect. Immun. 82, 500–508 (2014). 24478066 10.1128/IAI.01078-13 PMC3911389

[65] R. Okumura, T. Kurakawa, T. Nakano, H. Kayama, M. Kinoshita, D. Motooka, K. Gotoh, T. Kimura, N. Kamiyama, T. Kusu, Y. Ueda, H. Wu, H. Iijima, S. Barman, H. Osawa, H. Matsuno, J. Nishimura, Y. Ohba, S. Nakamura, T. Iida, M. Yamamoto, E. Umemoto, K. Sano, K. Takeda, Lypd8 promotes the segregation of flagellated microbiota and colonic epithelia. Nature 532, 117–121 (2016). 27027293 10.1038/nature17406

[66] T. C. Cullender, B. Chassaing, A. Janzon, K. Kumar, C. E. Muller, J. J. Werner, L. T. Angenent, M. Elizabeth Bell, A. G. Hay, D. A. Peterson, J. Walter, M. Vijay-Kumar, A. T. Gewirtz, R. E. Ley, Innate and adaptive immunity interact to quench microbiome flagellar motility in the gut. Cell Host Microbe 14, 571–581 (2013). 24237702 10.1016/j.chom.2013.10.009 PMC3920589

[67] M. P. Leatham, S. J. Stevenson, E. J. Gauger, K. A. Krogfelt, J. J. Lins, T. L. Haddock, S. M. Autieri, T. Conway, P. S. Cohen, Mouse intestine selects nonmotile *flhDC* mutants of *Escherichia coli* MG1655 with increased colonizing ability and better utilization of carbon sources. Infect. Immun. 73, 8039–8049 (2005). 16299298 10.1128/IAI.73.12.8039-8049.2005 PMC1307065

[68] E. J. Gauger, M. P. Leatham, R. Mercado-Lubo, D. C. Laux, T. Conway, P. S. Cohen, Role of motility and the *flhDC* operon in *Escherichia coli* MG1655 colonization of the mouse intestine. Infect. Immun. 75, 3315–3324 (2007). 17438023 10.1128/IAI.00052-07 PMC1932950

[69] G. M. Birchenough, E. E. Nystrom, M. E. Johansson, G. C. Hansson, A sentinel goblet cell guards the colonic crypt by triggering Nlrp6-dependent Muc2 secretion. Science 352, 1535–1542 (2016). 27339979 10.1126/science.aaf7419 PMC5148821

[70] K. M. Wheeler, G. Carcamo-Oyarce, B. S. Turner, S. Dellos-Nolan, J. Y. Co, S. Lehoux, R. D. Cummings, D. J. Wozniak, K. Ribbeck, Mucin glycans attenuate the virulence of *Pseudomonas aeruginosa* in infection. Nat. Microbiol. 4, 2146–2154 (2019). 31611643 10.1038/s41564-019-0581-8 PMC7157942

[71] M. Caldara, R. S. Friedlander, N. L. Kavanaugh, J. Aizenberg, K. R. Foster, K. Ribbeck, Mucin biopolymers prevent bacterial aggregation by retaining cells in the free-swimming state. Curr. Biol. 22, 2325–2330 (2012). 23142047 10.1016/j.cub.2012.10.028 PMC3703787

[72] Q. Liang, C. Ma, S. M. Crowley, J. M. Allaire, X. Han, R. W. W. Chong, N. H. Packer, H. B. Yu, B. A. Vallance, Sialic acid plays a pivotal role in licensing *Citrobacter rodentium*’s transition from the intestinal lumen to a mucosal adherent niche. Proc. Natl. Acad. Sci. U.S.A. 120, e2301115120 (2023). 37399418 10.1073/pnas.2301115120 PMC10334811

[73] A. R. Pacheco, M. M. Curtis, J. M. Ritchie, D. Munera, M. K. Waldor, C. G. Moreira, V. Sperandio, Fucose sensing regulates bacterial intestinal colonization. Nature 492, 113–117 (2012). 23160491 10.1038/nature11623 PMC3518558

[74] C. J. Worby, H. L. Schreiber IV, T. J. Straub, L. R. van Dijk, R. A. Bronson, B. S. Olson, J. S. Pinkner, C. L. P. Obernuefemann, V. L. Muñoz, A. E. Paharik, P. N. Azimzadeh, B. J. Walker, C. A. Desjardins, W.-C. Chou, K. Bergeron, S. B. Chapman, A. Klim, A. L. Manson, T. J. Hannan, T. M. Hooton, A. L. Kau, H. H. Lai, K. W. Dodson, S. J. Hultgren, A. M. Earl, Longitudinal multi-omics analyses link gut microbiome dysbiosis with recurrent urinary tract infections in women. Nat. Microbiol. 7, 630–639 (2022). 35505248 10.1038/s41564-022-01107-x PMC9136705

[75] K. A. Datsenko, B. L. Wanner, One-step inactivation of chromosomal genes in *Escherichia coli* K-12 using PCR products. Proc. Natl. Acad. Sci. U.S.A. 97, 6640–6645 (2000). 10829079 10.1073/pnas.120163297 PMC18686

[76] K. J. Wright, P. C. Seed, S. J. Hultgren, Development of intracellular bacterial communities of uropathogenic *Escherichia coli* depends on type 1 pili. Cell. Microbiol. 9, 2230–2241 (2007). 17490405 10.1111/j.1462-5822.2007.00952.x

[77] B. P. Cormack, R. H. Valdivia, S. Falkow, FACS-optimized mutants of the green fluorescent protein (GFP). Gene 173, 33–38 (1996). 8707053 10.1016/0378-1119(95)00685-0

[78] V. Kalas, J. S. Pinkner, T. J. Hannan, M. E. Hibbing, K. W. Dodson, A. S. Holehouse, H. Zhang, N. H. Tolia, M. L. Gross, R. V. Pappu, J. Janetka, S. J. Hultgren, Evolutionary fine-tuning of conformational ensembles in FimH during host-pathogen interactions. Sci. Adv. 3, e1601944 (2017). 28246638 10.1126/sciadv.1601944 PMC5302871

[79] L. Mydock-McGrane, Z. Cusumano, Z. Han, J. Binkley, M. Kostakioti, T. Hannan, J. S. Pinkner, R. Klein, V. Kalas, J. Crowley, N. P. Rath, S. J. Hultgren, J. W. Janetka, Antivirulence *C*-mannosides as antibiotic-sparing, oral therapeutics for urinary tract infections. J. Med. Chem. 59, 9390–9408 (2016). 27689912 10.1021/acs.jmedchem.6b00948 PMC5087331

[80] H. Liang, D. Poncet, E. Seydoux, N. D. Rintala, M. M. Maciel Jr, S. Ruiz, M. T. Orr, The TLR4 agonist adjuvant SLA-SE promotes functional mucosal antibodies against a parenterally delivered ETEC vaccine. NPJ Vaccines 4, 8–15 (2019). 31149350 10.1038/s41541-019-0116-6 PMC6538625

[81] I. M. N. Wortel, S. Kim, A. Y. Liu, E. C. Ibarra, M. J. Miller, Listeria motility increases the efficiency of epithelial invasion during intestinal infection. PLOS Pathog. 18, e1011028 (2022). 36584235 10.1371/journal.ppat.1011028 PMC9836302

