# Supplementary material for: Mechanisms of uropathogenic E. coli mucosal association in the gastrointestinal tract
Source: Sci Adv. 2025 Jan 31;11(5):eadp7066. doi: 10.1126/sciadv.adp7066 (PMC11784811; doi:10.1126/sciadv.adp7066)
Supplement: Supplementary file 1 — Figs. S1 to S8 Table S1 Legends for movies S1 and S2 [file sciadv.adp7066_sm.pdf]

Science Advances
AAAS

Supplementary Materials for

Mechanisms of uropathogenic *E. coli* mucosal association in the gastrointestinal tract

Philippe N. Azimzadeh *et al.*

Corresponding author: Scott J. Hultgren, hultgren@wustl.edu

Sci. Adv. **11**, eadp7066 (2025)
DOI: 10.1126/sciadv.adp7066

The PDF file includes:

Figs. S1 to S8
Table S1
Legends for movies S1 and S2

Other Supplementary Material for this manuscript includes the following:

Movies S1 and S2

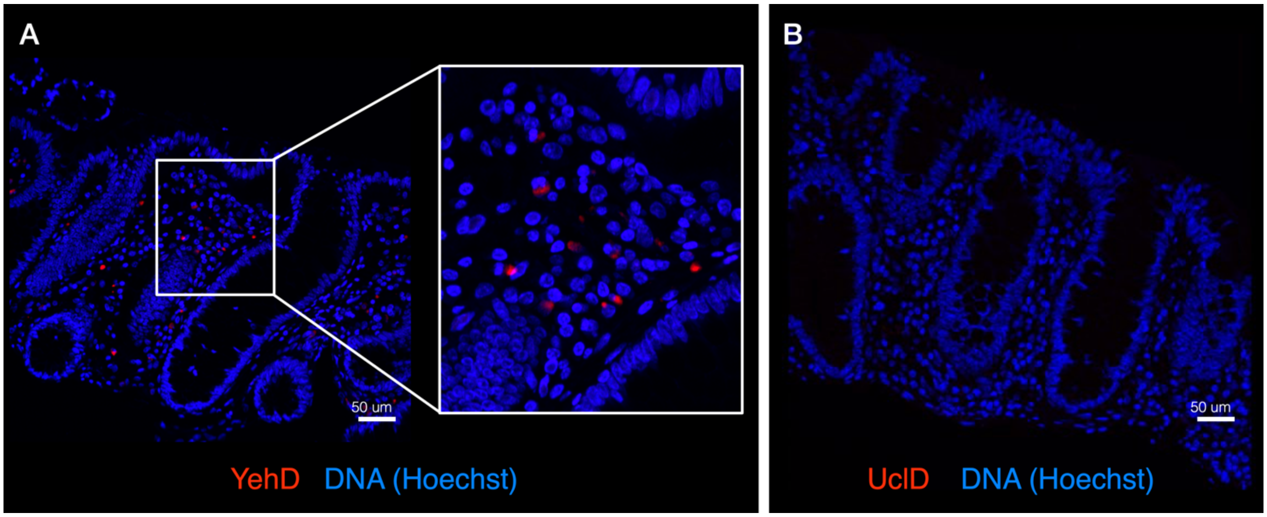

Figure S1: YehD and UclD lectins staining of PFA-fixed human colon sections. The purified and fluorescently labelled YehD and UclD lectin domains were bound to PFA-fixed human mucosal sections from healthy human colon tissue donors. Lectin domain are labelled in red, and DNA is labelled in blue (Hoechst stain). (A) YehD displays binding to unknown cells in the lamina propria, but does not bind epithelial tissue; n = 3 individuals, 2 sections each (B) UclD does not bind to human colon tissue; n = 3 individuals, 2 sections each.

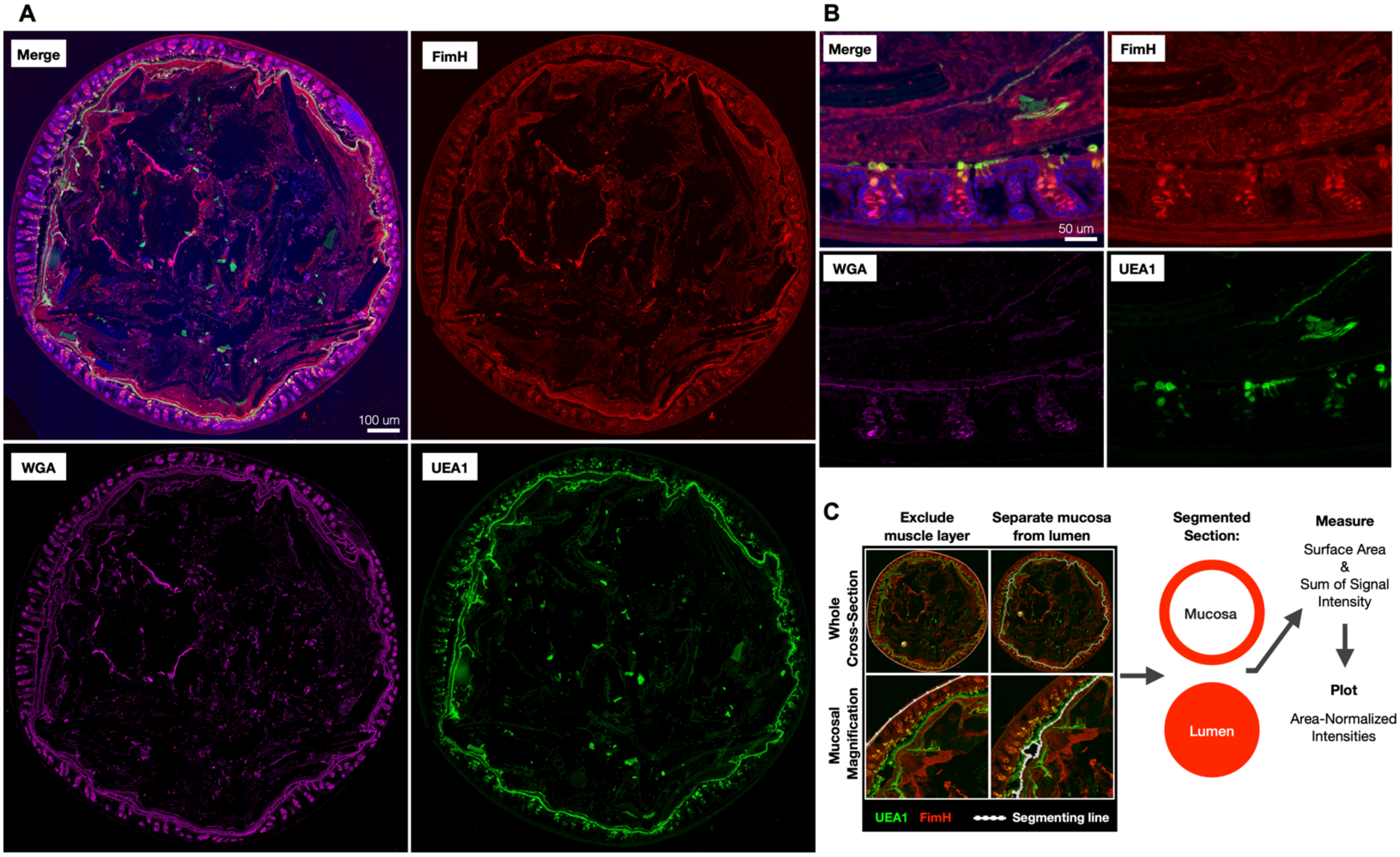

Figure S2: Additional counter-stained images of FimH mucosal binding and segmentation analysis strategy for lumen and mucosal signal intensity comparison. (A) Purified, fluorescently labelled FimH lectin domain was used to stain female C57BL/6NJ (Jackson) methacarn-fixed distal colon sections, and counter-stained with UEA1 (green) and WGA (pink) lectins. DNA is labelled by Hoechst counter-stain in blue. UEA1 specifically labels fucose residues, while WGA labels GlcNAc. Fucose is highly abundant in secrete mucus layers and pre-secretion crypt goblet cells. GlcNAc is abundant throughout secreted mucin as well as on the epithelium and in luminal (fecal) contents. Tile scans were generated to image whole mouse colon cross-sections. Related to Figure 1. **(B)** Mucosal magnification of the same colon section as in Fig. S2A. Related to Figure 2. FimH (red), UEA1 (green) WGA (pink), Hoechst (blue). **(C)** Segmentation strategy used to quantify and compare the FimH binding signal in the mucosal vs. luminal compartment. Segmentation lines were drawn manually using the surfaces function of Imaris 9 software, guided by the Hoechst and UEA1 stains.

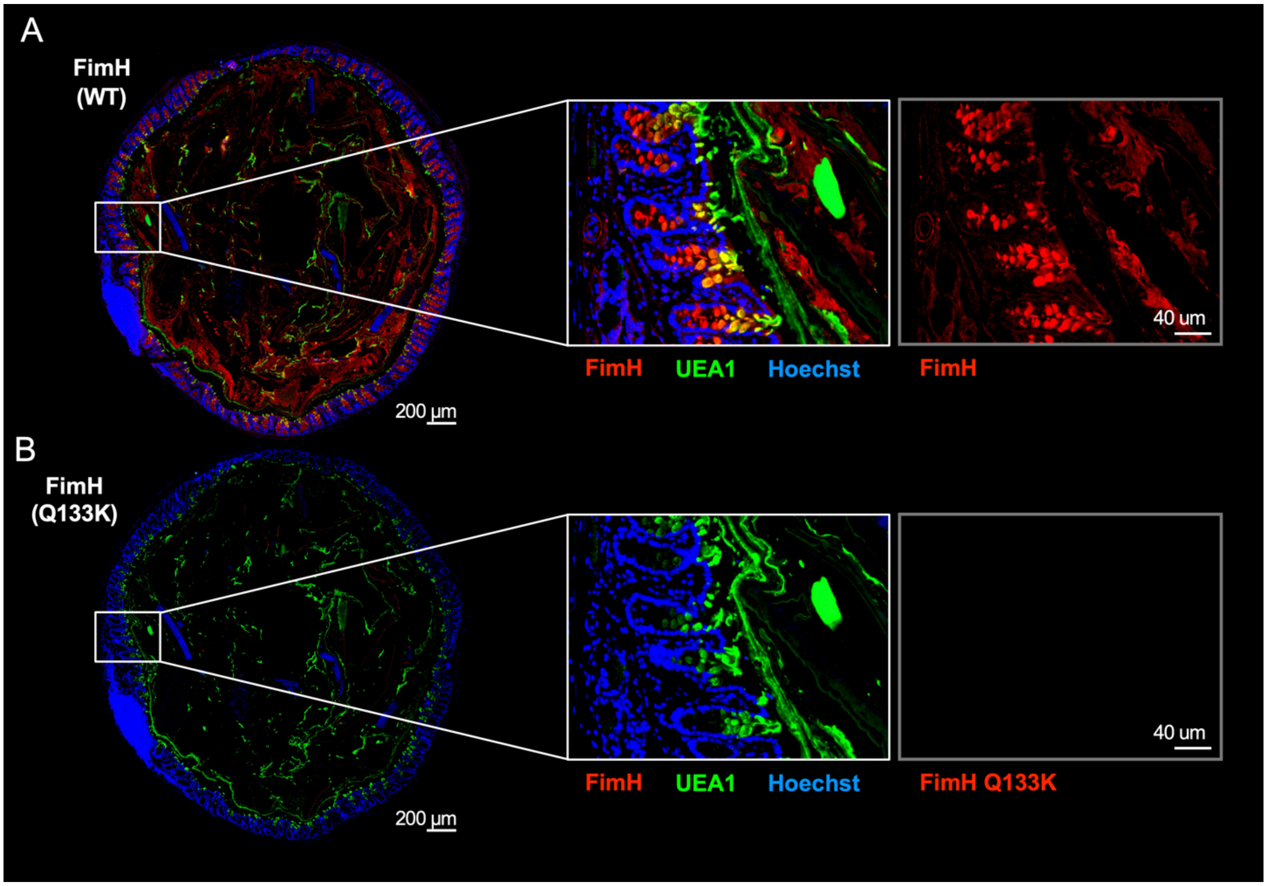

Figure S3 - Purified FimH::Q133K lectin domain does not bind the mouse colon. Binding of purified WT and Q133K mutant FimH lectin domain to mouse distal colon sections was compared. On the left of each panel is a representative tile scan of the entire colon cross-section, and on the right is a magnified view of the colon mucosa. Left scale bar = 200 µm, right scale bar = 100 µm. (A) WT FimH lectin domain is labelled in red, and DNA is labelled in blue (Hoechst stain). (B). FimH::Q133K mutant lectin domain is labelled in red, and DNA is labelled in blue (Hoechst stain). (A, B) n = 2 mice, 2 sections tested per mouse.

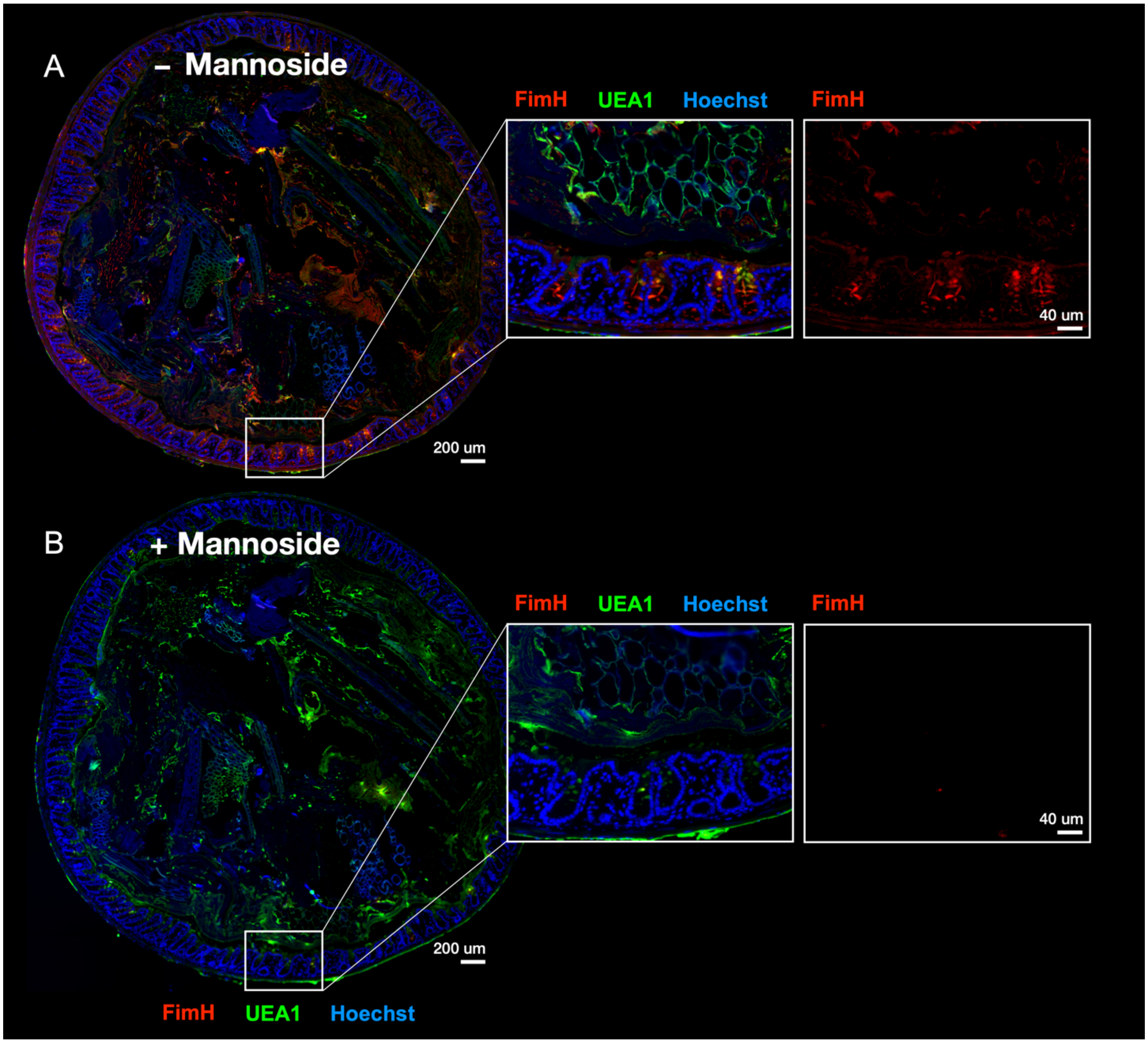

Figure S4 - Mannoside FIM1033 in 10X molar excess completely blocks FimH binding to mouse colon. On the left of each panel is a representative tile scan of the entire colon section, and on the right is a magnified view of the colon mucosa. Left scale bar = 100 µm, right scale bar = 20 µm. WT FimH lectin domain is labelled in red, UEA1 lectin is labelled in green, and DNA is labelled in blue (Hoechst stain). **(A)** Purified FimH lectin domain was incubated with PBS prior to staining mouse colon sections. **(B)** Purified FimH lectin domain was incubated with 10X molar excess of mannoside FIM1033 in PBS prior to staining mouse colon sections. **(A, B)** n = 2 mice, 2 sections tested per mouse.

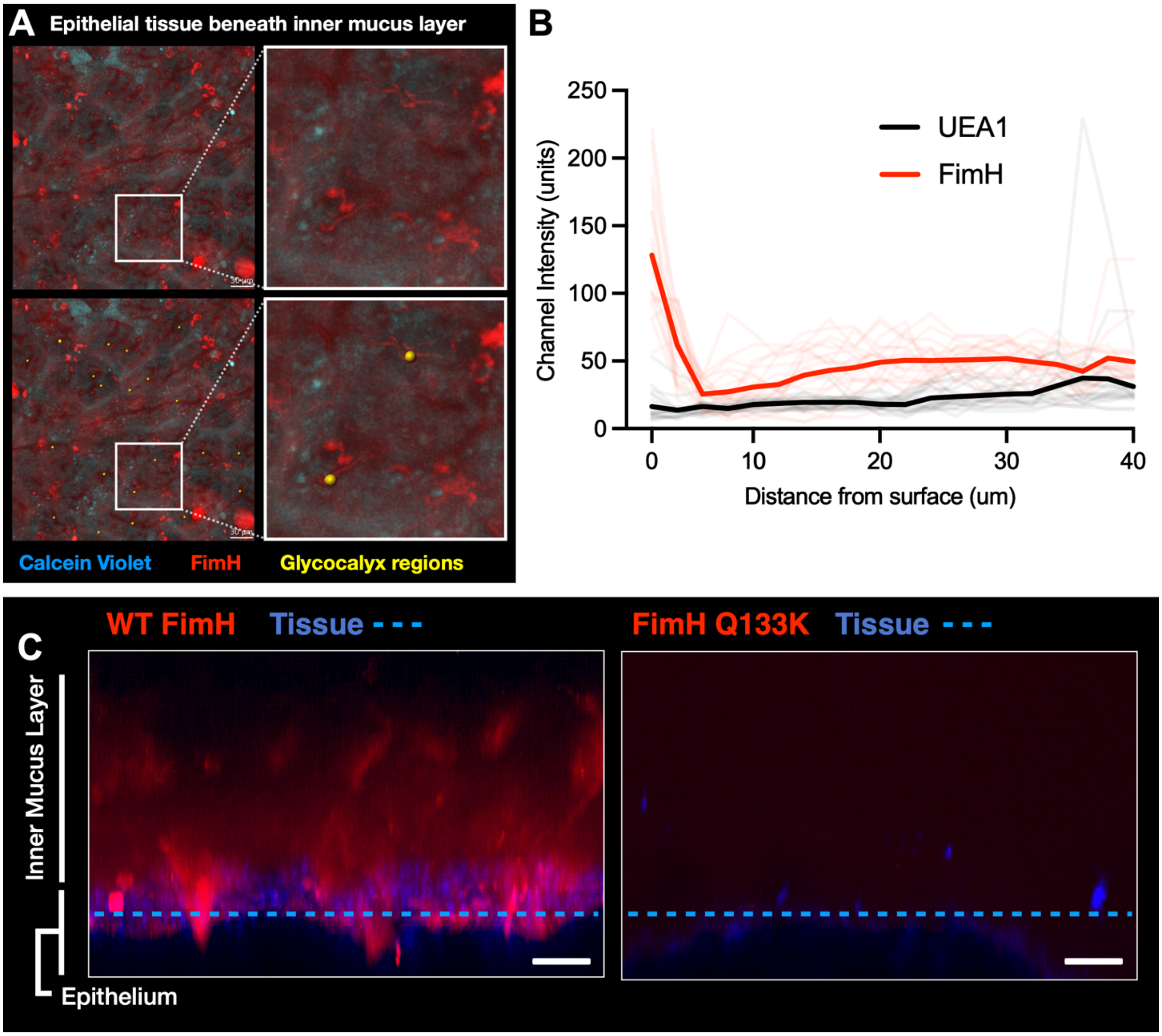

Figure S5 - WT FimH protein displays strong binding on glycocalyx regions at colon crypt entrances. Fluorescently labelled FimH (red) and UEA1 lectins were used to stain the IML *ex vivo* mouse colon preparations, with tissue stained by Calcein Violet. **(A)** Using Imaris 9 software, measurement spots were placed i) on glycocalyx regions at the mouth of crypt entrances of explanted colon tissue and ii) 40 µm above the first spot, in the Z-direction. **(B)** Intensity profiles of FimH (red) and UEA1 (black) from the crypt mouth to the middle of the IML layer. Mean shown in bold, and individual readings shown in pale color. **(C)** Representative side views (x/z projections) of the *ex vivo* mouse distal colon inner mucus layer (IML) reconstructed from confocal z-stacks (as in Figure 3). IML was stained with purified fluorescently labelled WT FimH lectin domain or FimH::Q133K mutant lectin domain. **(A, B)** n = 2 mice, with 2 pieces of colon tested per mouse. Dotted blue line denotes the top of the epithelial tissue, which was detected by capturing the second harmonic from two-photon imaging. Scale bar is 50 µm.

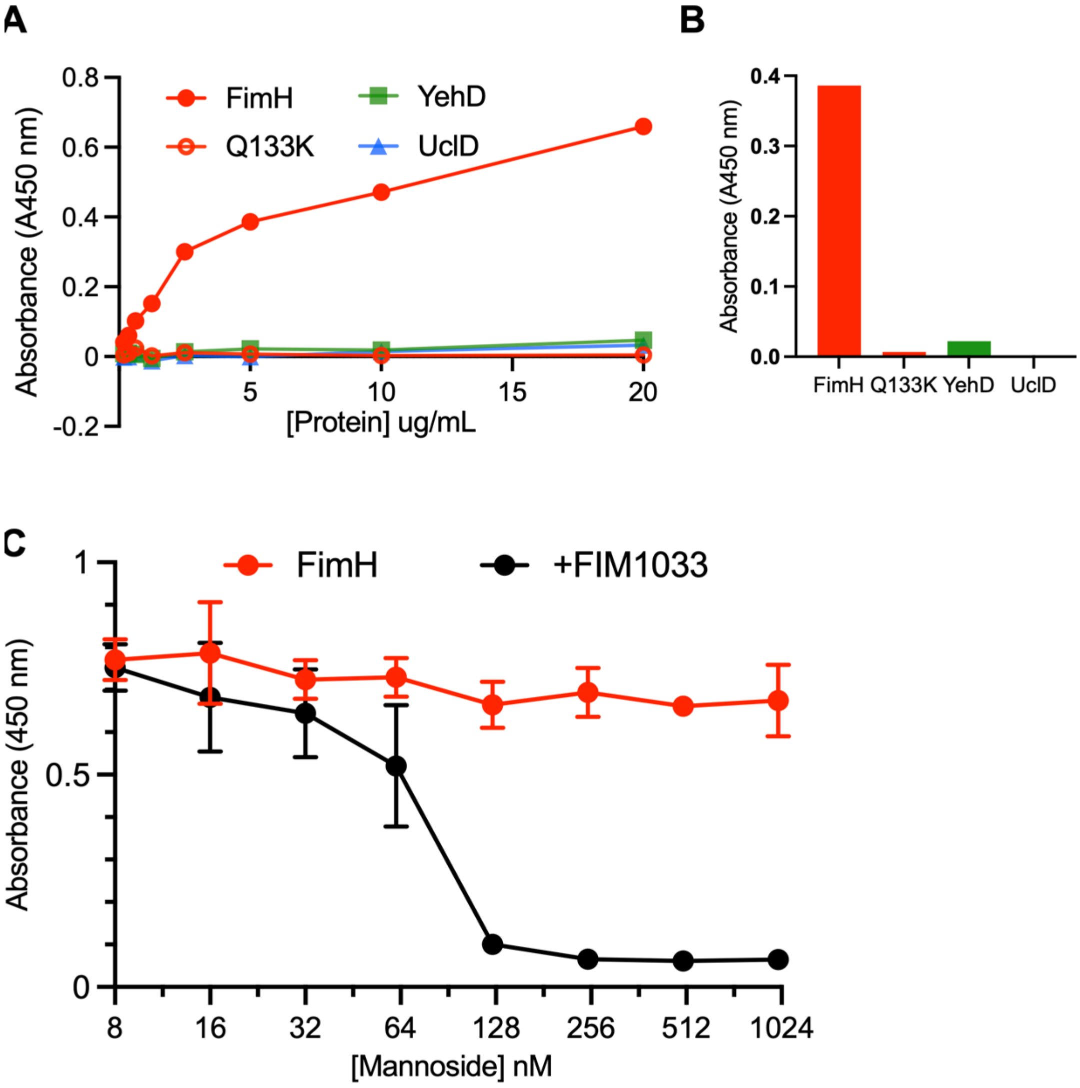

Figure S6 - Specificity of FimH for mucin in lectin domain binding assays with Bovine Submaxillary Mucin. (A) Biotinylated FimH-WT, FimH::Q133K, YehD, and UclD lectin domains binding to Bovine Submaxillary Mucin (BSM) displayed in protein dilution series (10 ug BSM adhered to plate). **(B)** Absorbance values for each lectin domain at 5 ug/mL of protein. **(C)** Absorbance readings from mannoside inhibition assay. 5 ug/mL of protein was added in all wells and mannoside compound FIM1033 was serially diluted from 1 uM down to 8 nM.

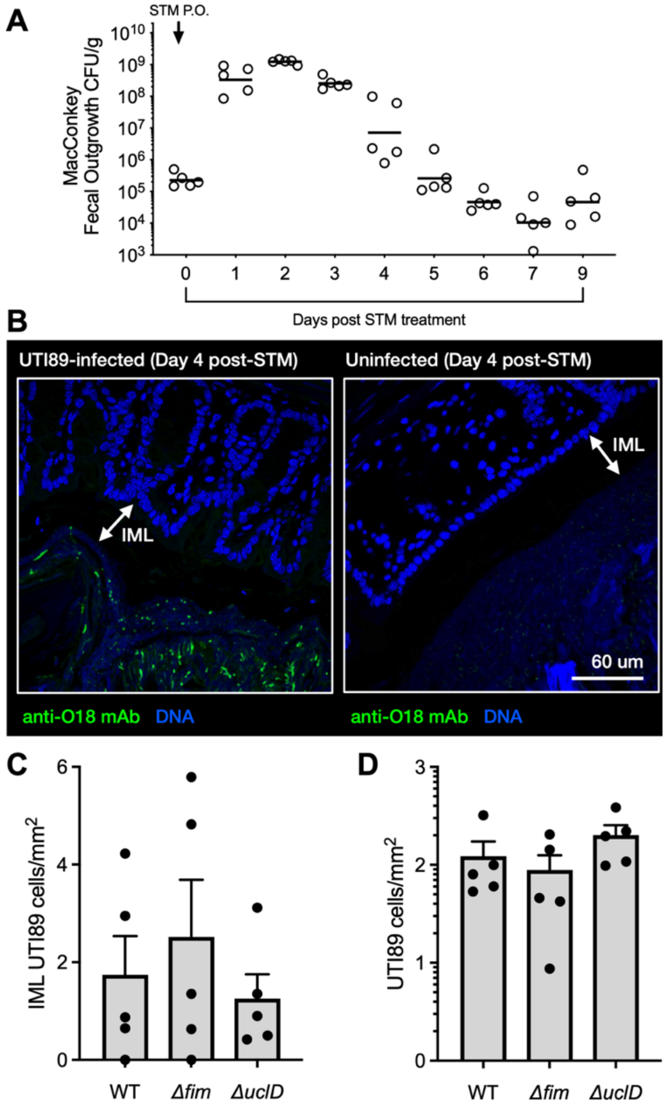

Figure S7: Anti-O18 LPS antibody specifically identifies UTI89 in mouse colon sections for analysis of colon biogeography. (A) Five female C3H/HeN mice (Envigo) where administered streptomycin (STM) in 100 uL of water via oral gavage (P.O.). Fecal pellets were collected immediately and daily for 9 days, and subsequently homogenized and plated onto MacConkey to quantify Enterobacteriaceae present in mice. (B) Twenty female C3H/HeN mice (Envigo) were treated with STM P.O. and gavaged with 10^8 of UTI89 mutants in PBS, or PBS control (100 uL) 24 hours later. Four days after UTI89 inoculation, mice were sacrificed, colons harvested, and fixed in methanol-Carnoy. Colons were subsequently paraffin embedded and sectioned to 5 um. Immunohistochemistry was performed using anti-O18 LPS primary antibody and Alexa488-conjugated secondary antibody, and fluorescence was subsequently imaged via confocal microscopy. Displayed are representative images from WT-colonized and uninfected control mice. UTI89 was not detected in uninfected controls. **(C)** Quantification of bacteria found within the inner mucus layer (IML) for each mutant (see Figure 4). **(D)** Total bacterial counts across all sections for each mutant (5 mice per group, 1 section per mouse).

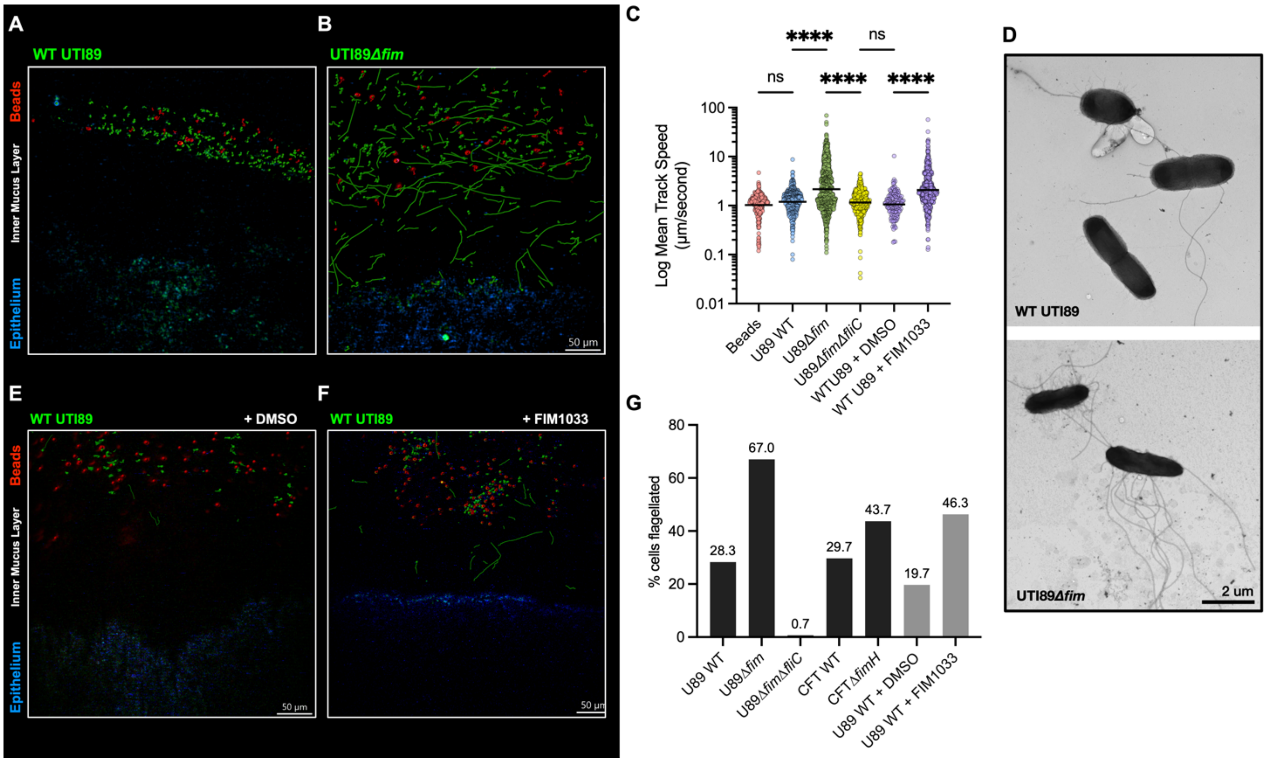

Figure S8 - Flagella mediated motility permits penetration of *ex vivo* mucus by UTI89*Δfim* and mannoside-treated WT UTI89. (A, B) A mixture of GFP-expressing WT UTI89 or UTI89*Δfim* and 1 µm fluorescent beads were added on top of ex vivo mouse colon preparations (see Figure 5). Time-lapse 2-photon microscopy was captured perpendicularly to the mucus layer at a fold in the colon tissue such that the inner mucus layer (IML) could be captured in the x/y projection plane (top-down imaging). Representative side views of the inner mucus layer (IML) reconstructed from confocal z-stacks are displayed on the left. See Figure M1 for a video of UTI89Δ*fim* motility. (E, F) GFP-expressing WT UTI89 was cultured in the presence of 10 mM mannoside or vehicle control (DMSO), and subsequently added on colon explants along with 1 um fluorescent beads. (A, B, E, F) Bacterial motility tracks are shown in green. Bead motility tracks are shown in red. Scale bar is 50 µm. (C) Quantification of the mean track speed of beads, UTI89 mutants, and WT UTI89 +/- mannoside. Each dot represents the mean track speed of one track. WT tracks were combined from two individual experiments (n=2), *Δfim* tracks were combined from three individual experiments (n=3), WT-DMSO tracks were combined from two experiments (n=2), WT-Mannoside tracks were combined from six experiments (n=3 mice). Bars represent the geometric mean. The Mann-Whitney U test was used to establish statistical significance between groups. ****p<0.00005. (D) UPEC strains were imaged by negative-stained Electron Microscopy in order to quantify flagellated cells. (G) Quantification of the percent of bacteria per mutant or culture condition which expressed flagella on the surface of the cell. 300 bacteria were counted per mutant or condition. "U89" refers to UTI89 and "CFT" refers to CFT073.

TABLE S1

Bacterial strains and plasmids

Strain or plasmid	Relevant genotype or features	Resistance(s)	Reference(s)
Strains			
UTI89 HK::Kan	Marked WT *E. coli* cystitis isolate	Kanamycin	(17)
UTI89*Δfim::Kan*	Type 1 replaced by KanR gene	Kanamycin	(75)
UTI89*Δfim* pcom-GFP	GFP+ FimH deletion strain	Kanamycin, Ampicillin	This work
UTI89*ΔfimΔfliC* pcom-GFP	GFP+ UTI89 lacking type 1 operon and FliC subunits	Kanamycin, Chloramphenicol, Ampicillin	This work
UTI89*ΔfliC*	UTI89Δ*fliC*::cmR	Chloramphenicol	This work
CFT073 HK::Kan	Marked WT *E. coli* cystitis isolate	Kanamycin	This work
CFT073*ΔfimH* pcom-GFP	CFT073 *ΔfimH*:*kanR*	Kanamycin, Ampicillin	This work
Plasmids			
pCom-GFP	Source of Ptac-gfp	Ampicillin	(77)

Movie S1 - Video microscopy of UTI89*Δfim* swimming through the inner mucus layer to reach the epithelium. GFP-expressing UTI89*Δfim* (green) and 1 um sized fluorescent beads (red) were added on top of distal mouse colon, as in Figure 5, and Figure S4a, right. Epithelial tissue is shown in blue. Two-photon microscopy was used to capture time-lapse images (approximately 2.5 frames per second) perpendicularly to the inner mucus layer at a fold of distal colon tissue. The video is played once without modification, and once with overalled motility tracking. Motility tracks for UTI89*Δfim* are shown in white, and motility tracks for beads are shown in yellow. Bacterial and bead motility were assessed by automated cell tracking (Bitplane Imaris). Scale bar = 50µm, time stamp = min:sec:msec (Lower right). The video is played back at 2.25x real-time.

Movie S2 - Video microscopy of UTI89*Δfim* within the epithelial crypts. GFP-expressing UTI89*Δfim* (green) and 1 um sized fluorescent beads (red) were added on top of expanded distal mouse colon, as in Figure 5. Epithelial tissue is shown in blue. Two-photon microscopy was used to capture time-lapse images in the x/y plane (a top top-down view of the epithelium). The video was captures at approximately one frame per second. The video is played once without modification, and once with overalled motility tracking. Motility tracks for UTI89*Δfim* are shown in white, and motility tracks for beads are shown in yellow. Bacterial motility was highlighted by automated cell tracking (Bitplane Imaris). Scale bar = 50 µm, time stamp = min:sec:msec (Lower right). The playback speed of movie S2 has been adjusted so that Supplemental Movie S1 and Movie S2 display time at the same rate (2.25x real-time).
